# Computational modelling of LY303511 and TRAIL-induced apoptosis suggests dynamic regulation of cFLIP

**DOI:** 10.1093/bioinformatics/bts702

**Published:** 2012-12-13

**Authors:** Yuan Shi, Gregory Mellier, Sinong Huang, Jacob White, Shazib Pervaiz, Lisa Tucker-Kellogg

**Affiliations:** ^1^Singapore-MIT Alliance, E4-04-10, 4 Engineering Drive 3, Singapore 117576, ^2^Duke-NUS, Graduate Medical School, National University of Singapore, Singapore 117597, ^3^Department of Physiology, National University of Singapore, 2 Medical Drive, Singapore 117597, ^4^Department of Electrical Engineering and Computer Science, M.I.T., Cambridge, MA 02139, USA, ^5^NUS Graduate School for Integrative Sciences and Engineering, Singapore 117456, ^6^Mechanobiology Institute, National University of Singapore, Singapore 117417 and ^7^Department of Dermatology, Stony Brook University, Stony Brook, NY 11794, USA

## Abstract

**Motivation:** TRAIL has been widely studied for the ability to kill cancer cells selectively, but its clinical usefulness has been hindered by the development of resistance. Multiple compounds have been identified that sensitize cancer cells to TRAIL-induced apoptosis. The drug LY303511 (LY30), combined with TRAIL, caused synergistic (greater than additive) killing of multiple cancer cell lines. We used mathematical modelling and ordinary differential equations to represent how LY30 and TRAIL individually affect HeLa cells, and to predict how the combined treatment achieves synergy.

**Results:** Model-based predictions were compared with *in vitro* experiments. The combination treatment model was successful at mimicking the synergistic levels of cell death caused by LY30 and TRAIL combined. However, there were significant failures of the model to mimic upstream activation at early time points, particularly the slope of caspase-8 activation. This flaw in the model led us to perform additional measurements of early caspase-8 activation. Surprisingly, caspase-8 exhibited a transient *decrease* in activity after LY30 treatment, prior to strong activation. cFLIP, an inhibitor of caspase-8 activation, was up-regulated briefly after 30 min of LY30 treatment, followed by a significant down-regulation over prolonged exposure. A further model suggested that LY30-induced fluctuation of cFLIP might result from tilting the ratio of two key species of reactive oxygen species (ROS), superoxide and hydrogen peroxide. Computational modelling extracted novel biological implications from measured dynamics, identified time intervals with unexplained effects, and clarified the non-monotonic effects of the drug LY30 on cFLIP during cancer cell apoptosis.

**Supplementary information:** Supplementary data are available at *Bioinformatics* online.

**Contact:** LisaTK@nus.edu.sg or Shazib_Pervaiz@nuhs.edu.sg

## 1 INTRODUCTION

Combinations of drugs can have non-obvious effects on a biological system, and the magnitude of their final effect can be synergistic, additive or antagonistic: meaning larger, the same as or smaller than the sum of the individual treatments (Chait *et al.*, 2007). For treating cancers, some of the current standards of care are combination therapies, but the development of synergistic combinations has been an expensive and laborious process of empirical testing (Fitzgerald *et al.*, 2006). Computational modelling of pathway dynamics has the potential to predict the effects of combination treatments, and could be useful in the design of multi-drug therapies. Single perturbations of a system (e.g. inhibiting an enzyme) are commonly studied using traditional experimental methods, but combinations of perturbations, too numerous to study experimentally, can be examined using computational tools (Koch *et al.*, 2009), based on existing models that describe the effects of single drugs. We used modelling to study the combination of TRAIL and LY303511, towards understanding their synergy and also aiding in the discovery of an unknown effect of LY303511.

The polypeptide ligand TRAIL (TNF-related apoptosis-inducing ligand, or Apo2L) induces death receptor-mediated apoptosis (programmed cell death) according to a well-studied pathway (Falschlehner *et al.*, 2007). TRAIL is currently undergoing phase I/II clinical evaluation for a variety of cancers, and it holds real promise as a therapeutic strategy owing to its selective targeting of cancer cells while sparing normal tissues (Abdulghani and El-Deiry, 2010; Amm *et al.*, 2011). However, development of resistance is a serious obstacle to the effective clinical use of TRAIL, and combination therapies to overcome resistance to TRAIL, or to induce re-sensitization to TRAIL, could be extremely important for enabling TRAIL-based therapies to succeed (Amm *et al.*, 2011; Lee *et al.*, 2007; Wang, 2008; Zhang and Fang, 2005).

The small molecule compound LY30 (LY303511) has been shown to sensitize multiple cancer cell lines to TRAIL-induced apoptosis (Poh *et al.*, 2007; Shenoy *et al.*, 2009) and vincristine-induced apoptosis (Poh and Pervaiz, 2005). LY30 is an inactive analogue of the phosphoinositide 3-kinase (PI3K) inhibitor LY294002 (Gharbi *et al.*, 2007; Jacobs *et al.*, 2005), and is related to the non-specific kinase inhibitor quercetin (Davies *et al.*, 2000). LY30 produces high levels of ROS (reactive oxygen species), which may be particularly harmful to cancer cells that have high metabolic rates and oxidative stress. Our recent studies found a synergistic anti-cancer effect by combining LY30 and TRAIL (Poh and Pervaiz, 2005; Poh *et al.*, 2007; Shenoy *et al.*, 2009). Treating cells with low doses of TRAIL plus with low doses of LY30 caused robust activation of the apoptotic pathway, while treatment with either agent alone caused minimal death.

We undertook the task of modelling the effects of LY30 in the network of TRAIL-induced apoptosis, with the aim of evaluating whether the molecular effects known to be caused by LY30 were sufficient to explain the synergy of the LY30–TRAIL combination. We were successful in using modelling to recapitulate the synergistic levels of cell death that were observed. However, the simulated activation kinetics of pro-apoptotic caspase enzymes along the pathway showed poor correlation with experimental data. In particular, there were large mismatches between simulation and experiment at early time points, particularly for upstream regions of the pathway. This suggested further experiments focusing on caspase-8 activation shortly after treatment. New data revealed an even larger and more obvious mismatch between simulation and experimental data: specifically, LY30 treatment had a brief inhibitory effect on caspase-8, 30 min after treatment. Finally, we modelled two sources of ROS that might explain the effects of LY30 on caspase-8 activation, and that might be relevant for other anti-cancer drugs.

## 2 METHODS

### 2.1 Cell and treatments

HeLa was purchased from ATCC (Rockville, MD, USA) and maintained in DMEM supplemented with 10% FBS, 1% l-glutamine and 1% S-penicillin. HeLa cells were plated at 0.125 million cells/well in 24-well plates (and proportionally for other size plates) and grown overnight until 80% confluent. All treatments with LY30 (Alexis, Switzerland) and TRAIL (Biomol, Plymouth Meeting, PA, USA) used the methods and doses of (Poh *et al.*, 2007); all combination treatments of LY30 and TRAIL involved pre-incubation of cells with 25 µM LY30 for 1 h before adding 20 ng/ml TRAIL. Experiments with the superoxide scavenger Tiron (Sigma-Aldrich) applied 10 mM to cells 1 h before adding LY30. Experiments with the H_2_O_2_ scavenger catalase (C3511 catalase from bovine liver, Sigma-Aldrich) used a dose of 2000 units/ml, added 1 day before treatment, then added again the next day for co-incubation with LY30 after changing the medium. Treatments for supplementary experiments are described in Supplementary Material 4.1.

### 2.2 Viability

After treatment with LY30, TRAIL or the combination for 24 h, cells were washed with 1× PBS, stained with crystal violet for 20 min, and washed three times with pure water. For quantification, crystal violet was dissolved in 1% SDS with shaking for 1 h, and then measured using absorbance at 595 nm with a Tecan microplate reader.

### 2.3 Caspase activity

HeLa cells, after the indicated treatments and incubations, were harvested, washed with 1× PBS, re-suspended in chilled cell lysis buffer (BD Pharmingen, San Diego, CA, USA) and incubated on ice for 10 min. Caspase-3 and Caspase-8 enzyme activities were assayed using 7-amino trifluoromethylcoumarin and 7-amino-4-methylcoumarin-conjugated substrates (BioMol) as reported previously (Poh *et al.*, 2007).

### 2.4 SDS-PAGE and western blotting

Hela cells were grown in 60 mm Petri dishes until 80% confluent, and treated with LY30 for the indicated durations. Cells were harvested and washed once with 1× PBS before lysis using cell lysis buffer (150 mM NaCl, Tris–HCl 7.4 and 1% Nonidet P40) with a cocktail of protease inhibiors (1 mM PMSF, 10 µg/ml aprotinin, 20 µg/ml pepstatin A and 10 µg/ml leupeptin). 100 µg of cell lysate was then subjected to SDS-PAGE on a 12% polyacrylamide gel before being electro-transferred onto Immobilon-P membranes (Millipore Corporation, Bedford, MA, USA). Membranes were blocked using 5% non-fat dry milk in TBST (TBS with 0.5% Tween20) and probed overnight at 4°C with cFLIP antibody (Santa Cruz Biotechnology Inc., Santa Cruz, CA, USA). β-actin or GAPDH (both from Santa Cruz Biotechnology Inc., Santa Cruz, CA, USA) was used as a loading control. Primary antibodies were detected using HRP-conjugated anti-mouse or anti-rabbit antibodies and visualized using enhanced chemiluminescence detection (ECL reagents from Roche, Indianapolis, IN, USA). Densities were quantified using Image J (http://rsb.info.nih.gov/ij/).

### 2.5 Simulations

All biological events were modelled with ordinary differential equations (ODEs) as elementary reactions with mass action kinetics. KroneckerBio toolbox (Toettcher *et al.*, 2010) in MATLAB (Polking, 1995) and Copasi (Hoops *et al.*, 2006) were used for simulating the ODE models. In the TRAIL pathway model, synthesis and degradation effects were only modelled for caspases. Degradation processes used first-order reactions, and protein synthesis used zeroth-order reactions, with synthesis rate assigned so that the initial concentrations of the Albeck model (Albeck *et al.*, 2008b) would be equal to the steady state of the model system without TRAIL. Monte Carlo simulations were carried out with sample size of 10 000 cells with normal distributions of initial concentrations (mean equal to the initial concentration in the Albeck model, and variance equal to 40% of the mean initial concentration). PLOT was used for visualizing simulations. The comparison of relative caspase activity measurements with simulations of absolute caspase numbers is described in Supplementary Material 1.6.

### 2.6 Statistical analysis

All experiments were performed at least three times for statistical significance. Numerical data were expressed as mean ± SD. Statistical analysis was performed using the one-tailed paired Student’s *t*-test considering the variances unequal. *P* values < 0.05 were considered significant.

## 3 RESULTS

### 3.1 Model construction for single and combination treatments

#### 3.1.1 Trail model 

**Fig. 1.**
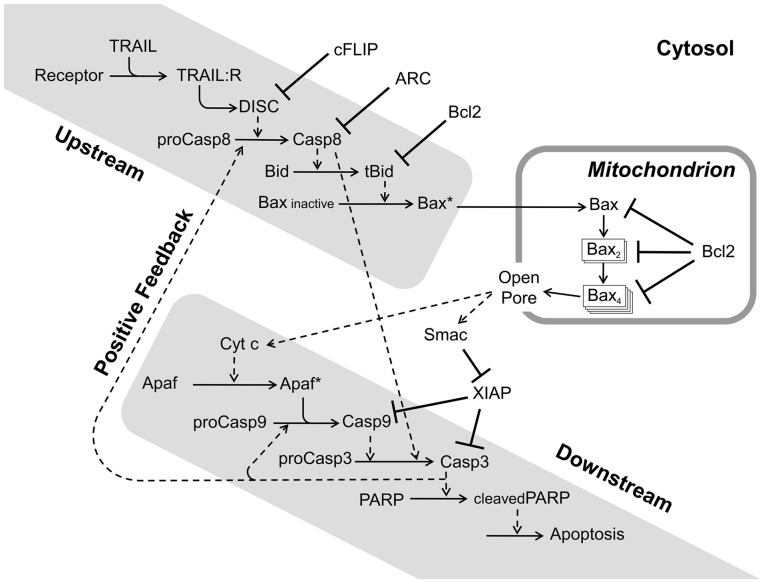
Schematic diagram of TRAIL-induced apoptosis. The dashed arrows indicate catalytic effects. Solid arrows indicate that the species at the base is consumed or translocated. For example, the oligomerization of mitochondrial Bax leads to formation of a pore in the mitochondrial outer membrane, which allows release of cytochrome c and Smac into the cytosol. Inhibitory relationships are denoted by a bar with crossbrace. (Details of inhibition are provided in Supplementary Tables S1.1–S1.3) Synthesis and degradation are not shown

To study the synergy between LY30 and TRAIL, we adapted a previous model of TRAIL-induced apoptosis (Albeck *et al.*, 2008b), and added reaction equations for the impact of LY30 on the network. The Albeck model was supported by extensive experimental measurements in HeLa (cervical carcinoma) cells, but those experiments involved cycloheximide, an inhibitor of protein synthesis. A lack of turnover effects in the Albeck model means that even an insignificant pro-apoptotic input is able to accumulate, without degradation, until the cell eventually dies (Bagci *et al.*, 2006; Eissing *et al.*, 2004). Apoptosis has evolved to occur in an all-or-nothing manner because one of the key outcomes of apoptosis is fragmentation of the DNA. Partial apoptosis would pose a grave danger to chromosomal integrity. To permit the system to simulate a bistable switch between survival and apoptosis, we included synthesis and degradation, by approximating them with mass-action rate equations (Fig. 1 and Supplementary Tables S1.1–S1.3). This modification renders the model less quantitative, but we believe this level of simplification is still highly informative for inferring system-level effects. We made several additional modifications to Albeck’s TRAIL model: feedback from activated caspase-3 to activated caspase-9 (Zou *et al.*, 2003), feedback from caspase-3 to caspase-8 without caspase-6 (Ferreira *et al.*, 2012; Yang *et al.*, 2006) and a higher concentration for the protein cFLIP (cellular FADD-like interleukin-1β-converting enzyme inhibitory protein), which was artificially lowered in the Albeck model as a side-effect of using cycloheximide (Kreuz *et al.*, 2001). Also we used Apoptosis Repressor with a Caspase recruitment domain (Heikaus *et al.*, 2008; Jo *et al.*, 2004) instead of bifunctional apoptosis regulator (BAR) to better represent the inhibition of caspase-8, which was stipulated by (Eissing *et al.*, 2004).

#### 3.1.2 LY30 model 

**Fig. 2.**
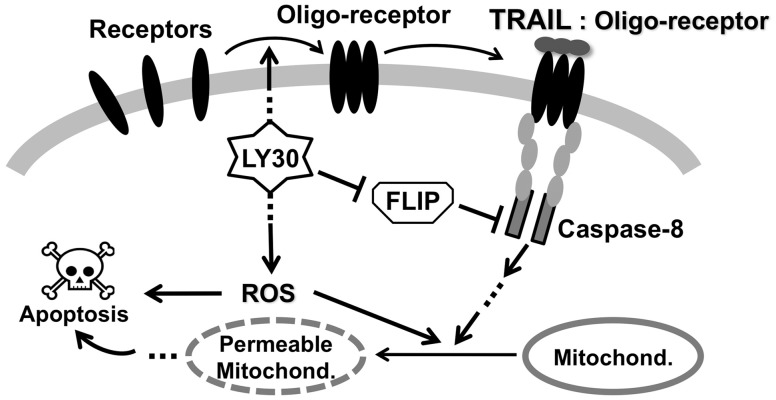
Schematic of how LY30 affects TRAIL-induced apoptosis. The receptors alone would have slower reaction rates than the primed receptors. Parameter values are listed in the Supplementary Material

Poh *et al.* identified two specific effects of LY30 that might explain its ability to sensitize HeLa carcinoma cells to TRAIL (Poh *et al.*, 2007): (i) clustering of TRAIL receptors, and (ii) down-regulating the pro-survival protein cFLIP. Figure 2 illustrates how these effects have been modelled. Clustering is modelled by an LY30-catalysed transition in the TRAIL receptor, converting a slow-reacting form into a faster-reacting form that we call ‘Primed-receptor’. The cFLIP down-regulation is modelled as an LY30-catalysed degradation reaction, which is intended to represent any variety of possible mechanisms including transcriptional repression, ubiquitylation, etc. In addition, LY30 has been shown to produce ROS (Poh and Pervaiz, 2005; Poh *et al.*, 2007), particularly hydrogen peroxide (H_2_O_2_) (Shenoy *et al.*, 2009), which can promote death through a variety of pathways (Clément and Pervaiz, 2001; Pervaiz and Clément, 2002). Here, we model ROS as causing some amount of mitochondrial permeability (Yamakawa *et al.*, 2000; Zuo *et al.*, 2009), and also causing mitochondria-independent death in a small subpopulation of cells (Hampton and Orrenius, 1997; Ruffels *et al.*, 2004). The parameter estimation is described in Supplementary Material 1.4. Our work is specific to TRAIL-induced apoptosis in HeLa cells, and we did not model additional phenomena found in other cell types (Shenoy *et al.*, 2009) or in other death pathways (Kristof *et al.*, 2005).

#### 3.1.3 LY30+trail model 

The TRAIL model was combined with the LY30 model to produce a theoretical model of how the combination of LY30 and TRAIL would affect apoptosis in HeLa. To mimic the combined treatment, we followed the experimental protocol of Poh *et al.*, pre-incubating cells with LY30 at 25 µM for 1 h before treating with TRAIL. In other words, the initial concentration of TRAIL was set to zero during the first hour with LY30 input, and then the TRAIL level was increased to the delivered dose of 20 ng/ml.

### 3.2 Simulation of synergistic effects

**Fig. 3.**
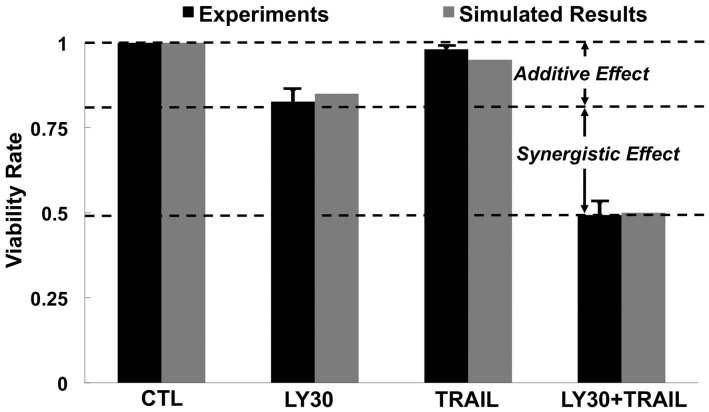
Simulated and observed cell viability. Cell viability was measured by crystal violet assay at 24 h after treatment with LY30 and/or TRAIL (repeated three times and normalized to untreated control)

Our previous measurements of apoptotic signalling in LY30-treated HeLa cells (Poh *et al.*, 2007) showed that the combination of LY30 and TRAIL induced synergistic (greater than additive) activation of many stages of the apoptotic pathway, including initiator and executioner caspases. To increase the statistical significance of the cell death measurements, we repeated the measurements of cell death. The combined dataset (Fig. 3) shows cell death to occur synergistically, with the rate of killing by TRAIL+LY30 to be 30% higher than the rate expected from a purely additive effect.

Apoptosis proceeds in an all-or-nothing fashion, as a ‘snap action’ switch, preceded by a variable time delay (Albeck *et al.*, 2008a; Albeck *et al.*, 2008b). This creates significant discrepancy between the concentrations in individual cells, and in the average concentrations in a population of cells that undergo apoptosis with variable delay. Individual cells exhibit much sharper slopes in their activation levels than the population average (Albeck *et al.*, 2008a). Because our model will be compared with data from populations of cells (immunoblots and enzyme activity assays), we need to model apoptosis signalling in a population of cells (Supplementary Fig. S1.5). We used Monte Carlo sampling to compute an average trajectory, using simulations of 10 000 instances for each treatment condition, and normally distributed initial concentrations with 40% variance. In keeping with previous work, we define apoptosis (death) to occur when poly ADP ribose polymerase is at least 50% cleaved (Albeck *et al.*, 2008b). Cell viability was simulated for single and combination drug treatments.

Comparing simulations against the Figure 3 experimental measurements showed the model to be successful at recapitulating the observed synergy between LY30 and TRAIL. This finding provides a ‘proof of plausibility’ that the LY30-induced effects of death receptor clustering, cFLIP down-regulation and ROS production are sufficient to facilitate significant sensitization to apoptosis, in cells treated with otherwise sub-lethal doses of TRAIL.

#### 3.2.1 Model discrepancy 

**Fig. 4.**
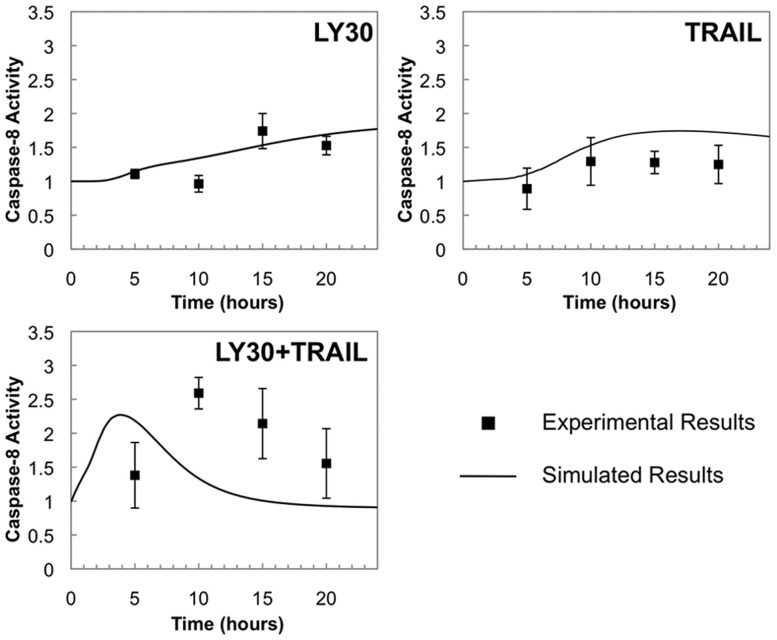
Comparison of simulated caspase-8 activity versus experimental measurements for caspase-8 activation by LY30 and TRAIL. Solid lines represent averaged results of 10 000 Monte Carlo simulations. Caspase-8 activity is plotted as relative fold-change versus untreated, meaning that untreated cells (time = 0) have activity 1.0. Black squares show the published fold-change of protein activity relative to untreated control. Supplementary Material 1.6 describes the conversion from simulated levels of absolute caspase-8 activity, into estimates of relative fold-change of measured activity, to account for cross-talk between multiple caspase isoforms

When we inspected the simulated levels of individual proteins, internal to the apoptotic pathway, we found the simulations did not resemble experimental observations. A variety of estimated parameter sets and model re-optimization efforts were unable to alleviate the qualitative divergence of the model from the observations (data not shown). Figure 4 compares simulated caspase-8 activity against experimentally measured caspase-8 activity for the same three treatment conditions. Simulations agreed reasonably with experiments for the treatments with LY30 alone or TRAIL alone. However, simulations predict that LY30+TRAIL induction of caspase-8 would achieve synergy (greater than additive) effect quickly and would peak before 5 h. In contrast, experimental measurements of caspase-8 showed a sharp rise much later (10 h) and no synergy at early time points. Mismatches were also seen downstream of caspase-8 (Supplementary Figs S1.7–S1.8). Because caspase activity measurements are not isoform specific, measurements were repeated using Bcl2 over-expression to decouple upstream and downstream caspases, but the caspase-8 trends were unchanged (Supplementary Figs S4.2–S4.5). A pattern of mismatch indicates a flaw in the model, and we hypothesize that biological events upstream of caspase-8 differ from the expected model, especially at early time points.

### 3.3 New experimental measurements of caspase 8 and cFLIP

The mismatch between model and experiments for the onset of LY30+TRAIL synergy provided a narrow specification for the molecules and time points in greatest need of clarification. New experiments were thus performed to measure caspase-8 activity at more finely spaced time points after LY30.

**Fig. 5.**
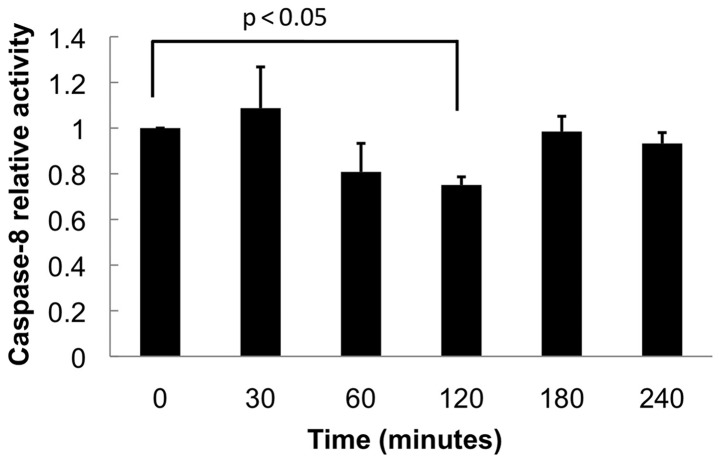
Caspase-8 activity measurements in Hela cells after different durations of LY30 treatment. Cells were treated with LY30 for 30, 60, 120, 180 or 240 min, or untreated (0 min). LY30 treatment caused a significant decrease of caspase-8 activity at 2 h, according to a one-sample *t*-test with Bonferroni correction

Figure 5 shows the effect of LY30 on caspase-8 enzymatic activity over time, with frequently spaced time points after treatment. We had expected LY30 to increase caspase-8 activity, but this experiment showed unexpected transient behaviours, with LY30 causing a significant decrease in caspase-8 activity at 2 h. Our model of LY30 effects (Fig. 2 and Supplementary Tables) does not include any possible way for LY30 to delay caspase-8 nor to inhibit apoptosis. This puzzle motivated us to perform more detailed measurements of caspase-8 dynamics.

**Fig. 6.**
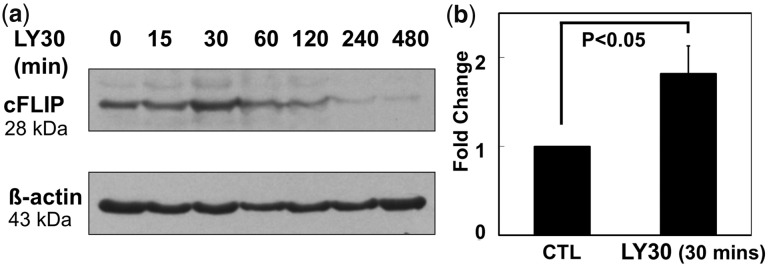
Western blot analysis of cFLIP in Hela after different durations of LY30 treatment. (**a**) Western blot of time dynamics of cFLIP after LY30 treatment; (**b**) Quantified fold-change of cFLIP protein levels after 30 min of LY30 treatment. cFLIP band intensities (with three biological replications) were normalized to β-actin intensity (loading control) before comparing with untreated to obtain relative fold-change

Some unknown effect of LY30 must occur, and we can infer it to be upstream of caspase-8, at least as soon as 2 h. Knowing that LY30 affects cFLIP (Poh *et al.*, 2007), and knowing that cFLIP has complex regulation (Fukazawa *et al.*, 2001; Peter, 2004), we repeated the measurements of cFLIP with denser time intervals. Figure 6 shows more complex dynamics of cFLIP protein expression. Surprisingly, cFLIP was initially up-regulated by LY30 treatment, before it declined. FADD levels were unchanged (Supplementary Fig. S4.7).

These up-and-down results demonstrated that the effect of LY30 on cFLIP is more complex than a simple down-regulation. To find a pathway to explain the non-monotonic regulation of cFLIP by LY30, we need to consider upstream regulators of cFLIP and downstream effectors of LY30. We next studied ROS effects in greater detail because ROS are known to be produced by LY30 (Poh and Pervaiz, 2005; Poh *et al.*, 2007; Shenoy *et al.*, 2009) as demonstrated by fluorescent measurements of dichlorofluorescin diacetate (DCFDA). Meanwhile, ROS is known to regulate cFLIP (Lee *et al.*, 2009).

DCFDA is the most commonly used indicator to measure ROS, but it can be activated by multiple types of ROS. ROS is a family of several species, with H_2_O_2_ (hydrogen peroxide) and 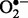 (superoxide) being the most abundant. In many cases, the ratio of 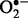 to H_2_O_2_ determines whether ROS will promote or hinder apoptosis (Clément and Pervaiz, 2001; Pervaiz and Clement, 2007; Pervaiz and Clément, 2002; Tang *et al.*, 2011). A high ratio of 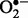 to H_2_O_2_ antagonizes apoptosis by triggering pro-survival pathways such as PI3K/Akt and extracellular signal-regulated kinase (ERK) (Lim and Clement, 2007; Zhu *et al.*, 2011). In contrast, a low ratio of 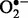 to H_2_O_2_ promotes apoptosis through intracellular acidification (Pervaiz and Clément, 2002), activation of caspase-3 and -9 (Yamakawa *et al.*, 2000), down-regulation of cFLIP (Nitobe *et al.*, 2003) and down-regulation of NHE1 (Akram *et al.*, 2006; Pervaiz and Clement, 2007).

### 3.4 New model of LY30-induced cFLIP regulation via ROS

**Fig. 7.**
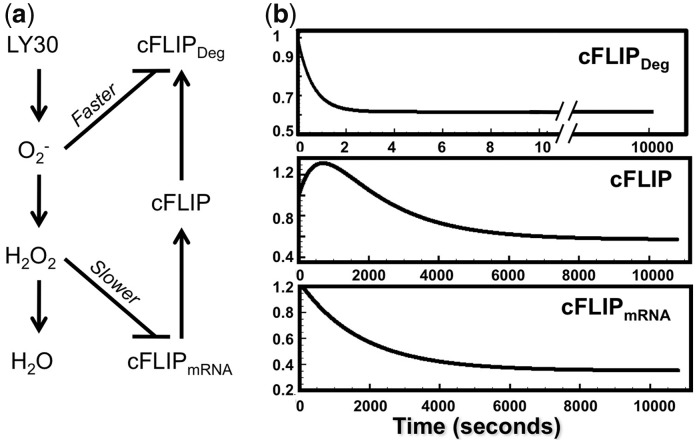
Hypothetical model for LY30 to cause non-monotonic regulation of cFLIP via 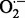 and H_2_O_2_. (**a**) Simplified diagram of LY30’s influence on cFLIP. This model is roughly divided into two phases. In the earlier phase, 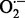 is produced after LY30 treatment, and the increased 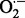 will block the degradation of cFLIP, thus inducing its up-regulation. In the later phase, H_2_O_2_ is produced by conversion of 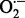, and inhibits the production of cFLIP, lowering its concentration. (**b**) Simulations of cFLIP, cFLIP_mRNA and degraded cFLIP (cFLIP_Deg) over time, as predicted by the model in Figure 7a. The model is fully specified in Supplementary Tables

We constructed a simple hypothetical model of 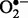 and H_2_O_2_ production and degradation, emphasizing the differential effects of 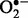 and H_2_O_2_ on cFLIP (Fig. 7a). Experimental evidence indicates that intracellular ROS and reactive nitrogen species (RNS) regulate cFLIP expression: nitric oxide (NO)-dependent S-nitrosylation of cFLIP prevents its ubiquitination (Iyer *et al.*, 2008), and S-nitrosylation can be augmented by superoxide. Conversely, H_2_O_2_ promotes ubiquitination and proteasomal degradation of cFLIP (Nitobe *et al.*, 2003), and H_2_O_2_ can cause a decrease in cFLIP expression. These cFLIP-related effects are consistent with our recent findings on the differential effects of intracellular H_2_O_2_ and 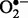 on cell fate signalling (Clément and Pervaiz, 2001; Pervaiz and Clément, 2002). The trend of 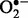 promoting survival and H_2_O_2_ promoting apoptosis was modelled in Figure 7a via cFLIP. This model includes cFLIP production, cFLIP degradation, inhibition of cFLIP degradation by 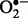, inhibition of cFLIP production by H_2_O_2_, as well as 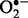 production, 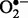 conversion into H_2_O_2_ and H_2_O_2_ degradation. (Reaction equations, parameters and literature sources are specified in Supplementary Tables S2.1–S2.3).

Our model of ROS and cFLIP was then simulated to explore whether the dynamics of 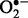 and H_2_O_2_ could plausibly explain both the cFLIP increase at 30 min and the cFLIP decrease at 6 h. Many compounds can cause production of 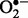 , which then gets converted into H_2_O_2_, so we designed the model to be generally applicable to ROS-producing anti-cancer drugs. Simulations (Fig. 7b) show that LY30-induced ROS could cause cFLIP levels to rise at 30 min and then fall in subsequent hours, due to faster pro-cFLIP influences from 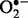, and slower anti-cFLIP influences from H_2_O_2_. Up-and-down behaviour of cFLIP can introduce delay into the apoptotic pathway (Supplementary Fig. S3.1), which explains why caspase-8 measurements did not jump up immediately after combination treatment. However, the early up-and-down trajectory was not sufficient to explain the later behaviour of caspase-8 (e.g. 10 h). Additional factors may contribute to the regulation of caspase-8. It is also possible that our measurements may be skewed by non-specific measurements, or by stochastic effects in the population (Brennan *et al.*, 2012).

**Fig. 8.**
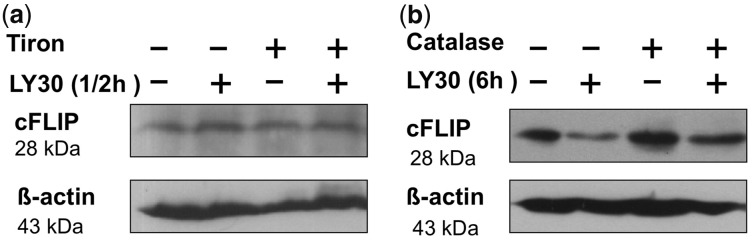
Western blots of cFLIP in Hela after LY30 treatment in the presence of ROS scavengers. (**a**) Western blot of cFLIP after 30 min LY30 treatment in the absence/presence of Tiron. Hela is pre-incubated with Tiron 1 h before adding LY30. (**b**) Western blot of cFLIP after 6 h LY30 treatment in the absence/presence of catalase

Finally, we performed preliminary experiments to explore model-based hypotheses from Figure 7, using anti-oxidant treatments (ROS scavengers) that are specific to certain sub-types of ROS (Supplementary Fig. S4.8). If LY30 is causing cFLIP to go up at 30 min via 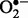, then removing 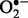 via Tiron treatment would be expected to halt the ability of LY30 to raise cFLIP at 30 min. Figure 8a shows that LY30 failed to increase cFLIP at 30 min when Tiron was present. If LY30 is causing later levels of cFLIP to go down via H_2_O_2_, then selectively removing H_2_O_2_ by adding catalase would restore cFLIP levels to the same as untreated. Figure 8b shows that cFLIP at 6 h after treatment with both LY30 and catalase had the same relative intensity as in untreated cells. The system of mechanisms in the Figure 7 model is plausible and will be the subject of future LY30 studies.

## 4 DISCUSSION AND CONCLUSION

An ODE model was constructed for combining the effects of two anti-cancer compounds, LY30 and TRAIL. The TRAIL effects were adapted from a previously published model (Albeck *et al.*, 2008b), and the LY30 effects were approximated from our previously published experiments on LY30 (Poh and Pervaiz, 2005; Poh *et al.*, 2007; Shenoy *et al.*, 2009). Some effects of LY30 are common to multiple drugs, and our model was designed to be maximally generic for similar cases. For example, the standard chemotherapeutic drugs doxorubicin and cisplatin both cause ROS production and cFLIP down-regulation. Also, resveratrol, the widely studied compound in red wine, causes ROS production and cFLIP down-regulation (Ding *et al.*, 2011; Ivanov *et al.*, 2008; Juan *et al.*, 2008). The ability of ROS to affect cFLIP is enormously important for determining whether cells are vulnerable to apoptosis (Geserick *et al.*, 2008), because multiple TRAIL-resistant cancers have shown re-sensitization to TRAIL after cFLIP levels were lowered (Hietakangas *et al.*, 2003; Kim *et al.*, 2008; Lee *et al.*, 2009; Nitobe *et al.*, 2003).

Every protein signalling network has abundant uncertainty, because every drug can have undiscovered effects, and every time-series experiment can have undiscovered peaks or dips occurring between the observed time points. Most such effects are minor, but some are significant enough to disrupt our ability to reason about system behaviour. Any modelling research, in addition to its primary goal, can thus be considered to have an implicit surveillance function of checking the consistency of the ‘known’ facts. In building the initial model of LY30 effects, we interpolated from a few measured time points of cFLIP, to obtain a simple approximation of LY30-induced decay of cFLIP, using a constant degradation rate (Supplementary Material 1.4). When the LY30 and TRAIL effects were combined, our inability to simulate the observed dynamics of caspase-8 suggested an error in the model. Our initial model had performed simplistic interpolation to simulate how LY30 affects cFLIP. We performed subsequent experiments that showed a non-monotonic ‘up-and-down’ concentration of cFLIP after treatment with LY30. These effects do not contradict the previous findings, but rather refine the kinetics. The new observations of cFLIP explain the delayed activation of caspase-8 and the delayed onset of synergy in the execution of apoptosis, because significant concentrations of cFLIP inhibit the activation of caspase-8 (Krueger *et al.*, 2001). Previous studies have used kinetic models of signalling pathways to detect mismatches between models and observations (Eissing *et al.*, 2004; Smieja *et al.*, 2008), but this work is among very few that have used pathway modelling to guide experiments towards actually confirming a previously unknown phenomenon (Hua *et al.*, 2005).

The remaining question was how LY30 causes non-monotonic effects on cFLIP. One simple explanation would be LY30 triggers two opposing pathways that regulate cFLIP in opposite ways. We built a model of ‘typical’ ROS production and degradation (Fig. 7a), including the known ability of H_2_O_2_ to cause gradual down-regulation of cFLIP (Nitobe *et al.*, 2003), and a hypothetical opposite effect of 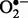 on cFLIP. In this model, the fundamental upstream–downstream relationship between 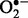 and H_2_O_2_, combined with the fast effects of 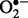 versus the slow-acting effects of H_2_O_2_, would together cause a time difference between the 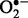-dominant phase (early cFLIP increase) and the H_2_O_2_-dominant phase (late cFLIP decline) of ROS-mediated effects. We performed immunoblots for the plausibility of this model, by blocking ROS species and testing whether LY30-induced changes in cFLIP were disrupted. Our model of cFLIP regulation was not falsified by these tests, and future work can perform more comprehensive experiments to characterize and validate the effects of LY30-induced 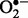 and H_2_O_2_ on cFLIP.

If LY30 is unique in causing complex cFLIP dynamics, it may be of narrow significance, but our model describes a general dynamic of ROS homeostasis. LY30 is one of many possible triggers for ROS, which might then cause two opposing effects on cFLIP. The predictions of this model may be applicable to other ROS-producing drugs, and to other redox-regulated protein levels. Future work can determine whether the same dynamics occur with doxorubicin, cisplatin and resveratrol. The timing of cell vulnerability to apoptosis (‘sensitization’) may also provide benefits for the design of optimal schedules for multi-drug treatments.

## Supplementary Material

Supplementary Data

## References

[bts702-B1] Abdulghani J, El-Deiry WS (2010). TRAIL receptor signaling and therapeutics. Expert Opin. Ther. Targets.

[bts702-B2] Akram S (2006). Reactive oxygen species-mediated regulation of the Na+-H+
exchanger 1 gene expression connects intracellular redox status with cells' sensitivity
to death triggers. Cell Death Differ..

[bts702-B3] Albeck JG (2008a). Quantitative analysis of pathways controlling extrinsic apoptosis in single
cells. Mol. cell.

[bts702-B4] Albeck JG (2008b). Modeling a snap-action, variable-delay switch controlling extrinsic cell
death. PLoS Biol..

[bts702-B5] Amm HM (2011). Combined modality therapy with TRAIL or agonistic death receptor
antibodies. Cancer Biol. Ther..

[bts702-B6] Bagci EZ (2006). Bistability in apoptosis: roles of bax, bcl-2, and mitochondrial
permeability transition pores. Biophys. J..

[bts702-B7] Brennan MD (2012). Systems biology. How information theory handles cell signaling and
uncertainty. Science.

[bts702-B8] Chait R (2007). Antibiotic interactions that select against resistance. Nature.

[bts702-B9] Clément MV, Pervaiz S (2001). Intracellular superoxide and hydrogen peroxide concentrations: a critical
balance that determines survival or death. Redox Rep..

[bts702-B10] Davies SP (2000). Specificity and mechanism of action of some commonly used protein kinase
inhibitors. Biochem. J..

[bts702-B11] Ding L (2011). Cisplatin restores TRAIL apoptotic pathway in glioblastoma-derived stem
cells through up-regulation of DR5 and down-regulation of c-FLIP. Cancer Invest..

[bts702-B12] Eissing T (2004). Bistability analyses of a caspase activation model for receptor-induced
apoptosis. J. Biol. Chem..

[bts702-B13] Falschlehner C (2007). TRAIL signalling: decisions between life and death. Int. J. Biochem. Cell Biol..

[bts702-B14] Ferreira KS (2012). Caspase-3 feeds back on caspase-8, Bid and XIAP in type I Fas signaling in
primary mouse hepatocytes. Apoptosis.

[bts702-B15] Fitzgerald JB (2006). Systems biology and combination therapy in the quest for clinical
efficacy. Nat. Chem. Biol..

[bts702-B16] Fukazawa T (2001). Accelerated degradation of cellular FLIP protein through the
ubiquitin-proteasome pathway in p53-mediated apoptosis of human cancer
cells. Oncogene.

[bts702-B17] Geserick P (2008). Suppression of cFLIP is sufficient to sensitize human melanoma cells to
TRAIL- and CD95L-mediated apoptosis. Oncogene.

[bts702-B18] Gharbi SI (2007). Exploring the specificity of the PI3K family inhibitor
LY294002. Biochem. J..

[bts702-B19] Hampton MB, Orrenius S (1997). Dual regulation of caspase activity by hydrogen peroxide: implications for
apoptosis. FEBS Lett..

[bts702-B20] Heikaus S (2008). Caspase-8 and its inhibitors in RCCs *in vivo*: the
prominent role of ARC. Apoptosis.

[bts702-B21] Hietakangas V (2003). Erythroid differentiation sensitizes K562 leukemia cells to TRAIL-induced
apoptosis by downregulation of c-FLIP. Mol. Cell Biol..

[bts702-B22] Hoops S (2006). COPASI—a complex pathway simulator. Bioinformatics.

[bts702-B23] Hua F (2005). Effects of Bcl-2 levels on Fas signaling-induced caspase-3 activation:
molecular genetic tests of computational model predictions. J. Immunol..

[bts702-B24] Ivanov VN (2008). Resveratrol sensitizes melanomas to TRAIL through modulation of
antiapoptotic gene expression. Exp. Cell Res..

[bts702-B25] Iyer AK (2008). Role of S-nitrosylation in apoptosis resistance and
carcinogenesis. Nitric Oxide.

[bts702-B26] Jacobs MD (2005). Pim-1 ligand-bound structures reveal the mechanism of serine/threonine
kinase inhibition by LY294002. J. Biol. Chem..

[bts702-B27] Jo DG (2004). Calcium binding of ARC mediates regulation of caspase 8 and cell
death. Mol. Cell Biol..

[bts702-B28] Juan ME (2008). Resveratrol induces apoptosis through ROS-dependent mitochondria pathway in
HT-29 human colorectal carcinoma cells. J. Agric. Food Chem..

[bts702-B29] Kim YH (2008). Rosiglitazone promotes tumor necrosis factor-related apoptosis-inducing
ligand-induced apoptosis by reactive oxygen species-mediated up-regulation of death
receptor 5 and down-regulation of c-FLIP. Free Radic. Biol. Med..

[bts702-B30] Koch G (2009). Modeling of tumor growth and anticancer effects of combination
therapy. J. Pharmacokinet. Pharmacodyn..

[bts702-B31] Kreuz S (2001). NF-kappaB inducers upregulate cFLIP, a cycloheximide-sensitive inhibitor of
death receptor signaling. Mol. Cell. Biol..

[bts702-B32] Kristof AS (2005). LY303511 (2-piperazinyl-8-phenyl-4H-1-benzopyran-4-one) acts via
phosphatidylinositol 3-kinase-independent pathways to inhibit cell proliferation via
mammalian target of rapamycin (mTOR)- and non-mTOR-dependent mechanisms. J. Pharmacol. Exp. Ther..

[bts702-B33] Krueger A (2001). Cellular FLICE-inhibitory protein splice variants inhibit different steps
of caspase-8 activation at the CD95 death-inducing signaling complex. J. Biol. Chem..

[bts702-B34] Lee JY (2007). The NO TRAIL to YES TRAIL in cancer therapy (review). Int. J. Oncol..

[bts702-B35] Lee TJ (2009). Withaferin A sensitizes TRAIL-induced apoptosis through reactive oxygen
species-mediated up-regulation of death receptor 5 and down-regulation of
c-FLIP. Free Radic. Biol. Med..

[bts702-B36] Lim S, Clement MV (2007). Phosphorylation of the survival kinase Akt by superoxide is dependent on an
ascorbate-reversible oxidation of PTEN. Free Radic. Biol. Med..

[bts702-B37] Nitobe J (2003). Reactive oxygen species regulate FLICE inhibitory protein (FLIP) and
susceptibility to Fas-mediated apoptosis in cardiac myocytes. Cardiovasc. Res..

[bts702-B38] Pervaiz S, Clément MV (2002). A permissive apoptotic environment: function of a decrease in intracellular
superoxide anion and cytosolic acidification. Biochem. Biophys. Res. Commun..

[bts702-B39] Pervaiz S, Clement MV (2007). Superoxide anion: oncogenic reactive oxygen species?
*Int*. J. Biochem. Cell boil..

[bts702-B40] Peter ME (2004). The flip side of FLIP. Biochem. J..

[bts702-B41] Poh TW, Pervaiz S (2005). LY294002 and LY303511 sensitize tumor cells to drug-induced apoptosis via
intracellular hydrogen peroxide production independent of the phosphoinositide
3-kinase-Akt pathway. Cancer Res..

[bts702-B42] Poh TW (2007). LY303511 amplifies TRAIL-induced apoptosis in tumor cells by enhancing DR5
oligomerization, DISC assembly, and mitochondrial permeabilization. Cell Death Differ..

[bts702-B43] Polking JC (1995). MATLAB Manual for Ordinary Differential Equations.

[bts702-B44] Ruffels J (2004). Activation of ERK1/2, JNK and PKB by hydrogen peroxide in human
SH–SY5Y neuroblastoma cells: role of ERK1/2 in H2O2induced cell
death. Eur. J. Pharmacol..

[bts702-B45] Shenoy K (2009). LY303511 enhances TRAIL sensitivity of SHEP-1 neuroblastoma cells via
hydrogen peroxide-mediated mitogen-activated protein kinase activation and up-regulation
of death receptors. Cancer Res..

[bts702-B46] Smieja J (2008). Model-based analysis of interferon-beta induced signaling
pathway. Bioinformatics.

[bts702-B47] Tang H (2011). The scavenging of superoxide radicals promotes apoptosis induced by a novel
cell-permeable fusion protein, sTRAIL:FeSOD, in tumor necrosis factor-related
apoptosis-inducing ligand-resistant leukemia cells. BMC Biol..

[bts702-B48] Toettcher J, Peng L (2010). Recycling circuit simulation techniques for mass-action biochemical
kinetics. Simulation and Verification of Electronic and Biological Systems.

[bts702-B49] Wang S (2008). The promise of cancer therapeutics targeting the TNF-related
apoptosis-inducing ligand and TRAIL receptor pathway. Oncogene.

[bts702-B50] Yamakawa H (2000). Activation of caspase-9 and -3 during H2O2-induced apoptosis of PC12 cells
independent of ceramide formation. Neurol. Res..

[bts702-B51] Yang S (2006). Caspase-3 mediated feedback activation of apical caspases in doxorubicin
and TNF-alpha induced apoptosis. Apoptosis.

[bts702-B52] Zhang L, Fang B (2005). Mechanisms of resistance to TRAIL-induced apoptosis in
cancer. Cancer Gene Ther..

[bts702-B53] Zhu P (2011). Angiopoietin-like 4 protein elevates the prosurvival intracellular
O2(-):H2O2 ratio and confers anoikis resistance to tumors. Cancer Cell.

[bts702-B54] Zou H (2003). Regulation of the Apaf-1/caspase-9 apoptosome by caspase-3 and
XIAP. J. Biol. Chem..

[bts702-B55] Zuo Y (2009). Oxidative modification of caspase-9 facilitates its activation via
disulfide-mediated interaction with Apaf-1. Cell Res..

