# Supplementary material for: Computational modelling of LY303511 and TRAIL-induced apoptosis suggests dynamic regulation of cFLIP
Source: Bioinformatics. 2012 Dec 13;29(3):347–54. doi: 10.1093/bioinformatics/bts702 (PMC3562069; doi:10.1093/bioinformatics/bts702)
Supplement: Supplementary Data [file supp_29_3_347__index.html]

Computational Modeling of LY303511 and TRAIL-Induced Apoptosis Suggests Dynamic Regulation of cFLIP — Computational modelling of LY303511 and TRAIL-induced apoptosis suggests dynamic regulation of cFLIP — Supplementary Data 

# Computational modelling of LY303511 and TRAIL-induced apoptosis suggests dynamic regulation of cFLIP

## Supplementary Data

files

**Files in this Data Supplement:**

- Supplementary Data - pdf file
