# Supplementary material for: Computational modelling of LY303511 and TRAIL-induced apoptosis suggests dynamic regulation of cFLIP
Source: Bioinformatics. 2012 Dec 13;29(3):347–54. doi: 10.1093/bioinformatics/bts702 (PMC3562069; doi:10.1093/bioinformatics/bts702)
Supplement: Supplementary Data [file supp_bts702_Supplementary_Information.pdf]

# SUPPLEMENTARY INFORMATION

## "COMPUTATIONAL MODELING OF LY303511 AND TRAIL-INDUCED APOPTOSIS SUGGESTS DYNAMIC REGULATION OF cFLIP"

Yuan Shi, Gregory Mellier, Sinong Huang, Jacob White, Shazib Pervaiz and Lisa Tucker-Kellogg

**SUPPLEMENTARY INFORMATION 1**
**1. THE LY30+TRAIL MODEL 2**
Supplementary Table 1.1: Reaction Equations of the LY30+TRAIL model 2
Supplementary Table 1.2: Species and Initial Concentrations 4
Supplementary Table 1.3: Rate Constants 6
Supplementary Text 1.4: Parameter Estimation for the LY30 + TRAIL Model 8
Supplementary Figure 1.5: Single Cells versus Cell Populations (in silico) 11
Supplementary Text 1.6: Conversion of absolute activity into relative fold-change 12
Supplementary Figure 1.7: Caspase-3 activity in experiments and simulations. 13
Supplementary Figure 1.8: Simulated Trajectories of Species 14
**2. THE ROS-CFLIP MODEL 16**
Supplementary Table 2.1: Reactions of the ROS-cFLIP model 16
Supplementary Table 2.2: Initial Concentrations of the ROS-cFLIP Model 17
Supplementary Table 2.3: Reaction Rates of the ROS-cFLIP model 19
**3. THE TRAIL+ LY30 MODEL COMBINED WITH ROS-CFLIP 21**
Supplementary Figure 3.1: the TRAIL+ LY30 model combined with ROS-cFLIP 21
**4. BIOLOGICAL RESULTS 23**
Supplementary Text 4.1: Supplementary Experimental Methods 23
Supplementary Figure 4.2: Caspase-8 activity in HeLa with empty vector control 24
Supplementary Figure 4.3: Caspase-8 activity in HeLa with Bcl2 overexpression 25
Supplementary Figure 4.4: Caspase-3 activity in HeLa with empty vector control 26
Supplementary Figure 4.5: Caspase-3 activity in HeLa with Bcl2 overexpression 27
Supplementary Figure 4.6: LY30-induced changes in cFLIP levels 28
Supplementary Figure 4.7: FADD expression. 29
Supplementary Figure 4.8: Activity of ROS Scavengers 30
**5. REFERENCES 31**

# 1. The LY30+TRAIL Model

## *Supplementary Table 1.1: Reaction Equations of the LY30+TRAIL model*

$$TRAIL + R \xrightleftharpoons[k\_1]{k1} TRAIL:R \xrightarrow{KK1} R*$$

$$R*+cFLIP \xrightleftharpoons[k\_2]{k2} R*:cFLIP$$

$$R*+C8 \xrightleftharpoons[k\_3]{k3} R*:C8 \xrightarrow{KK3} R*+C8*$$

$$C8*+ARC \xrightleftharpoons[k\_4]{k4} C8*:ARC$$

$$C8*+C3 \xrightleftharpoons[k\_5]{k5} C8*:C3 \xrightarrow{KK5} C8*+C3*$$

$$C3*+C8 \xrightleftharpoons[k\_6]{k6} C3*:C8 \xrightarrow{KK6} C3*+C8*$$

$$C3*+XIAP \xrightleftharpoons[k\_8]{k8} C3*:XIAP \xrightarrow{KK8} XIAP$$

$$C3*+PARP \xrightleftharpoons[k9]{k9} C3*:PARP \xrightarrow{KK9} C3*+cPARP$$

$$C8*+Bid \xrightleftharpoons[k\_10]{k10} C8*:Bid \xrightarrow{KK10} C8*+tBid$$

$$tBid + Bcl2c \xrightleftharpoons[k\_11]{k11} tBid:Bcl2c$$

$$tBid + Bax \xrightleftharpoons[k\_12]{k12} tBid:Bax \xrightarrow{KK12} tBid + Bax*$$

$$Bax* \xrightleftharpoons[k\_13]{k13} Baxm*$$

$$Baxm*+Bcl2 \xrightleftharpoons[k\_14]{k14} Baxm*:Bcl2$$

$$Baxm*+Baxm* \xrightleftharpoons[k\_15]{k15} Bax2$$

$$Bax2 + Bcl2 \xrightleftharpoons[k\_16]{k16} Bax2:Bcl2$$

$$Bax2 + Bax2 \xrightleftharpoons[k\_17]{k17} Bax4$$

$$Bax4 + Bcl2 \xrightleftharpoons[k\_18]{k18} Bax4:Bcl2$$

$$Bax4 + M \xrightleftharpoons[k\_19]{k19} Bax4:M \xrightarrow{KK19} M*$$

$$M*+Cycm \xrightleftharpoons[k\_20]{k20} M*:Cycm \xrightarrow{KK20} M*+Cycr$$

$$M*+Smacm \xrightleftharpoons[k\_21]{k21} M*:Smacm \xrightarrow{KK21} M*+Smacr$$

$$Cycr \underset{k\_22}{\overset{k22}{\rightleftarrows}} Cyc$$

$$Cyc + Apaf \underset{k\_23}{\overset{k23}{\rightleftarrows}} Cyc : Apaf \xrightarrow{KK23} Cyc + Apaf *$$

$$Apaf * + C9 \underset{k\_24}{\overset{k24}{\rightleftarrows}} Apop$$

$$Apop + C3 \underset{k\_25}{\overset{k25}{\rightleftarrows}} Apop : C3 \xrightarrow{KK25} Apop + C3*$$

$$Smacr \underset{k\_26}{\overset{k26}{\rightleftarrows}} Smac$$

$$Apop + XIAP \underset{k\_27}{\overset{k27}{\rightleftarrows}} Apop : XIAP$$

$$XIAP + Smac \underset{k\_28}{\overset{k28}{\rightleftarrows}} Smac : XIAP$$

$$C8* \xrightarrow{KK29} \phi$$

$$C3* \xrightarrow{KK29} \phi$$

$$C6* \xrightarrow{KK29} \phi$$

$$Apop \xrightarrow{KK29} \phi$$

$$C8 \xrightarrow{KK30} \phi$$

$$C3 \xrightarrow{KK30} \phi$$

$$C9 \xrightarrow{KK30} \phi$$

$$\phi \xrightarrow{KK31} C8$$

$$\phi \xrightarrow{KK33} C3$$

$$\phi \xrightarrow{KK34} C9$$

$$LY30 \xrightarrow{k35} LY30 + ROS$$

$$ROS \underset{k\_36}{\overset{k36}{\rightleftarrows}} ROS *$$

$$LY30 + cFLIP \xrightarrow{k37} LY30$$

$$LY30 \xrightarrow{k38} \phi$$

$$LY30 + R \underset{k\_39}{\overset{k39}{\rightleftarrows}} LY30 : R \xrightarrow{KK39} LY30 + R\_primed$$

$$TRAIL + R\_primed \underset{k\_40}{\overset{k40}{\rightleftarrows}} TRAIL : R\_primed \xrightarrow{KK40} R*$$

$$C3* + C9 \underset{k\_41}{\overset{k41}{\rightleftarrows}} C3* : C9 \xrightarrow{KK41} Apop + C3*$$

$$ROS * + PARP \underset{K\_42}{\overset{k42}{\rightleftarrows}} ROS* : PARP \xrightarrow{KK42} ROS * + cPARP$$

$$ROS * + M \underset{k\_43}{\overset{k43}{\rightleftarrows}} ROS* : M \xrightarrow{KK43} ROS * + M *$$

*Supplementary Table 1.2: Species and Initial Concentrations*

| Species Name | I.C. (molecules/cell) | Description |
|---|---|---|
| TRAIL | 1200 | Death Ligand |
| R_primeed | 0 | Clustered form of receptor |
| R | 200 | Monomer form of receptor |
| L:R | 0 | Ligand-receptor complex, inactive form |
| R* | 0 | Death Inducing Signal Complex(DISC), active complex |
| FLIP | 4000 | cFLIP, inhibitor of caspase 8 activation |
| R*:flip | 0 | Complex of DISC with cFLIP |
| C8 | 20000 | pro-caspase 8, initiator caspase of extrinsic pathway |
| C8* | 0 | Activated caspase 8 (Activated caspases are in the form C<#>*) |
| ARC | 1000 | Apoptosis Repressor with Caspase Recruitment Domain, Inhibitor of C8* |
| C3 | 10000 | pro-caspase 3, inactive form |
| C6 | 10000 | pro-caspase 6, inactive form |
| XIAP | 100000 | X-linked Inhibitor of Apoptosis, targets C3* or C9* |
| PARP | 1000000 | DNA damage repair enzyme |
| cPARP | 0 | Cleaved PARP, indicative of Apoptosis |
| Bid | 40000 | Substrate of C8*, inactive form |
| tBid | 0 | Cleaved Bid, active form |
| Bcl2c | 20000 | Cytosolic Bcl2, inhibits Bid or Bax |
| Bax | 100000 | Target of tBid |
| Bcl2 | 20000 | Bcl2 in Mitochondria, can bind to Bax*, Bax2 or |

| | | |
|---|---|---|
| | | Bax4 |
| Bax2 | 0 | Dimer of Bax* |
| Bax4 | 0 | Tetramer of Bax* |
| M | 500000 | Mitochondrial membrane  closed pores |
| M* | 0 | Number of active pores on the outer membrane of the mitochondia |
| CyCm | 500000 | bound Cytochrome c inside mitochondria |
| CyCr | 0 | unbound Cytochrome c inside mitochondria |
| Smacm | 100000 | bound Smac inside mitochondria |
| Smacr | 0 | free Smac released in cytosol |
| CyC | 0 | free Cytochrome c in cytosol |
| Apaf | 100000 | Apaf-1, inactive form |
| Apaf* | 0 | Apaf-1, active form |
| C9 | 100000 | pro-caspase 9, inactive form |
| Apop | 0 | Apoptosome, Apaf*:C9 |
| Apop:C3 | 0 | Complex of pro-caspase 3 with Apoptosome |
| Smac | 0 | free Smac inside cytosol |
| LY30 | 4.1 | LY30, experimental compound that sensitizes cells to TRAIL |
| R_oligo | 10 | Oligomerized form of receptors |
| ROS | 0 | Reactive Oxygen Species, pro-form |
| ROS_active | 0 | Reactive Oxygen Species, active form |

In the table, *X** denotes the active form of an inactive protein *X*. For example , C8* is the active form of C8.

*Supplementary Table 1.3: Rate Constants*

| Index | Name | Value | Index | Name | Value | Index | Name | Value |
|---|---|---|---|---|---|---|---|---|
| 1 | k1 | 4.00E-07 | 43 | k_17 | 1.00E-03 | 85 | k_40 | 1.00E-3 |
| 2 | k_1 | 1.00E-03 | 44 | k18 | 1.00E-06 | 86 | KK40 | 3.00E+10 |
| 3 | KK1 | 1.00E-05 | 45 | k_18 | 1.00E-03 | 87 | k41 | 4E-09 |
| 4 | k2 | 1.00E-06 | 46 | k19 | 1.00E-06 | 88 | k_41 | 0.001 |
| 5 | k_2 | 1.00E-03 | 47 | k_19 | 1.00E-03 | 89 | KK41 | 1 |
| 6 | k3 | 1.00E-06 | 48 | KK19 | 1.00E+00 | 90 | k42 | 2E-6 |
| 7 | k_3 | 1.00E-03 | 49 | k20 | 2.00E-06 | 91 | k_42 | 0 |
| 8 | KK3 | 1 | 50 | k_20 | 1.00E-03 | 92 | KK42 | 1.2 |
| 9 | k4 | 1.00E-06 | 51 | KK20 | 1.00E+01 | 93 | k43 | 3E-10 |
| 10 | k_4 | 1.00E-03 | 52 | k21 | 2.00E-06 | 94 | k_43 | 0 |
| 11 | k5 | 1.00E-07 | 53 | k_21 | 1.00E-03 | 95 | KK43 | 0.005 |
| 12 | k_5 | 1.00E-03 | 54 | KK21 | 1.00E+01 | | | |
| 13 | KK5 | 1.00E+00 | 55 | k22 | 1.00E-02 | | | |
| 14 | k6 | 1.00E-06 | 56 | k_22 | 1.00E-02 | | | |
| 15 | k_6 | 1.13 | 57 | k23 | 5.00E-07 | | | |
| 16 | KK6 | 1.00E-01 | 58 | k_23 | 1.00E-03 | | | |
| 17 | k7 | 3.00E-08 | 59 | KK23 | 1.00E+00 | | | |
| 18 | k_7 | 1.00E-03 | 60 | k24 | 5.00E-08 | | | |
| 19 | KK7 | 1.00E+00 | 61 | k_24 | 1.00E-03 | | | |
| 20 | k8 | 2.00E-06 | 62 | k25 | 5.00E-09 | | | |
| 21 | k_8 | 1.00E-03 | 63 | k_25 | 1.00E-03 | | | |
| 22 | KK8 | 1.00E-01 | 64 | KK25 | 1.00E+00 | | | |
| 23 | k9 | 1.00E-06 | 65 | k26 | 1.00E-02 | | | |

| | | | | | | | | |
|---|---|---|---|---|---|---|---|---|
| 24 | k_9 | 1.00E-02 | 66 | k_26 | 1.00E-02 | | | |
| 25 | KK9 | 1.00E+00 | 67 | k27 | 2.00E-06 | | | |
| 26 | k10 | 1.00E-07 | 68 | k_27 | 1.00E-03 | | | |
| 27 | k_10 | 1.00E-03 | 69 | k28 | 7.00E-06 | | | |
| 28 | KK10 | 1.00E+00 | 70 | k_28 | 1.00E-03 | | | |
| 29 | k11 | 1.00E-06 | 71 | KK29 | 8.35E-04 | | | |
| 30 | k_11 | 1.00E-03 | 72 | KK30 | 7.23E-10 | | | |
| 31 | k12 | 1.00E-07 | 73 | KK31 | KK30*IC(C8) | | | |
| 32 | k_12 | 1.00E-03 | 74 | KK33 | KK30*IC(C3) | | | |
| 33 | KK12 | 1.00E+00 | 75 | KK34 | KK30*IC(C9) | | | |
| 34 | k13 | 1.00E-02 | 76 | k35 | 8.20E-7 | | | |
| 35 | k_13 | 1.00E-02 | 77 | k36 | 6.83E-8 | | | |
| 36 | k14 | 1.00E-06 | 78 | K37 | 2.05E-10 | | | |
| 37 | k_14 | 1.00E-03 | 79 | K38 | 5.00E-5 | | | |
| 38 | k15 | 1.00E-06 | 80 | K39 | 1.37E-7 | | | |
| 39 | k_15 | 1.00E-03 | 81 | K_39 | 1.00E-03 | | | |
| 40 | k16 | 1.00E-06 | 82 | KK39 | 8.30E-05 | | | |
| 41 | k_16 | 1.00E-03 | 83 | k40 | 8.60E-04 | | | |
| 42 | k17 | 1.00E-06 | 84 | | | | | |

*ki* rates are in particles/sec. *k_i* and *KKi* rates are in units of sec$^{-1}$. Parameters 1-70 are from the TRAIL model of (Albeck, et al., 2008). Parameters 71-10 were estimated using our previous experimental work (Poh, et al., 2007) as detailed below.

## *Supplementary Text 1.4: Parameter Estimation for the LY30 + TRAIL Model*

**LY30-induced Receptor Oligomerization.** Quantification of receptor oligomerization was conducted using our previous experiments that pulled down DR5 from HeLa lysate using excess or limited amounts of antibody (Poh et al., 2007). The total amount of DR5 protein was the same in LY30-treated cells and untreated, but the amount of DR5 pulled down by limited antibodies was 5.8-fold greater in LY30-treated cells than in untreated

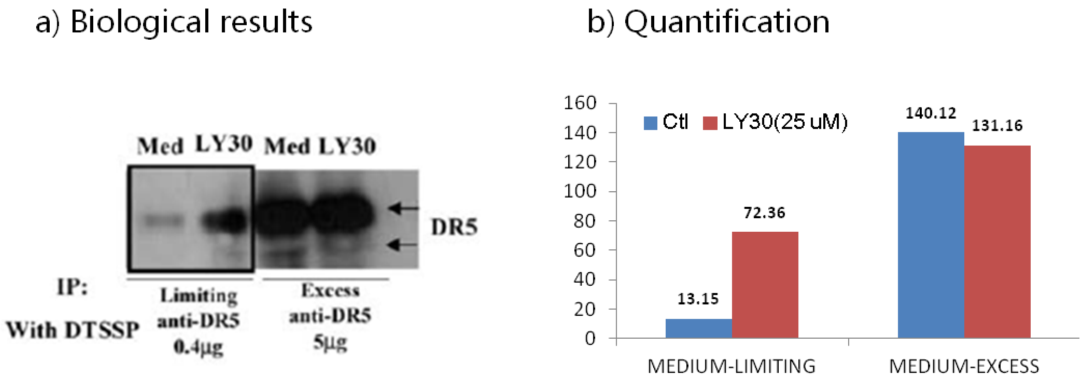


Supplementary Figure 1.4.1. LY30-induced receptor oligomerization. (a) Experimental results reprinted from (Poh. et al, 2007) and (b) quantified density of the DR5 bands using ImageJ.

cells (Supplementary Figure 1.4.1). If the pull-down ratio had been close to 3-fold, we might have characterized the transition as trimerization, because trimerization is receptor stoichiometry for activation. Instead we considered analogies to published results finding that clustered or oligomerized or lipid-raft-localized receptors transduce TRAIL signals more readily than non-clustered receptors (Mellier and Pervaiz, 2012). For example, the DR clustering induced by LY30 resembles the lipid raft localization of DRs, induced by the ROS-producing compound resveratrol (Delmas, et al., 2004). Therefore, we modeled DR5 oligomerization as a change of state. In untreated cells, all receptors were assumed to be initially an unprimed state *R*. We then modeled that receptor interaction with LY30 would trigger a state transition towards a cluster-localized state called *R_primed*.

$$R + LY30 \underset{k\_39}{\overset{k39}{\rightleftarrows}} R:LY30 \xrightarrow{KK39} R\_primed + LY30$$

The rate parameters of this reaction were chosen so that an initial dose of R would reach steady state levels of R_primed at approximately 1 hour, and also so that the R:LY30 complex would have low concentration.

**DISC formation.** For DISC formation, we retained the rates (*k1, k_1, KK1)* from Albeck et al. for unprimed receptors. For primed receptors, *k40* was set to 1e-4 and and *k_40*

$$R\_primed + TRAIL \underset{k\_40}{\overset{k40}{\rightleftarrows}} R\_primed:TRAIL \xrightarrow{KK40} R^*$$

Was set to 1e-3, the same as Albeck et al. We used the following to estimate the final degree of freedom, the *KK40* reaction for activation of the primed receptors After 5 minutes of exposure to TRAIL, immuno-precipitation of the FADD-containing complex with DR5 (the DISC-like complex) showed roughly 7-fold more assembly of DISC-like complexes in cells pre-treated with LY30, compared with cells that had not been pre-treated. See Supplementary Figure 1.4.2.

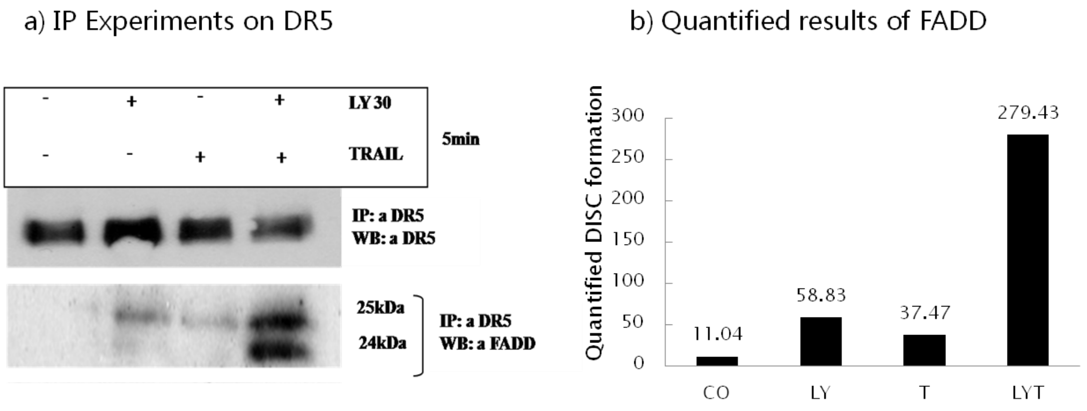


Supplementary Figure 1.4.2 Time kinetics of DISC assembly upon LY30+TRAIL treatment. (a) Western blot of FADD through immuno-precipitated with anti-DR5 in HeLa cells. HeLa cells were exposed to TRAIL (20ng/ml) for 5 min with or without prior addition of LY30(25µM) for one hr (Poh, et al.). (b) Quantification of FADD from Western blots.

The existing model was simulated to obtain the level of R* at 5 minutes after treatment with TRAIL alone. The experimentally measured fold-change in DISC-like complex formation at 5 minutes was used to compute an expected fold-change increase in R* for simulations of the LY+T treatment at 5 minutes. Then the KK40 parameter was adjusted so that simulations of LY+T would achieve the expected value of R* at 5 minutes.

**Doses.** We stimulated the model with 1200 molecules/cell of TRAIL to represent 20ng/ml of TRAIL, consistent with prior use of 3000 molecules/cell to represent 50ng/ml of TRAIL. The dose of LY30 was not calibrated to physical units, and the dose-response of LY30 was not studied. Therefore, simulations of LY30 should either use zero dose, or else use the same unitless dose (4.1) that we used when calibrating the simulated downstream effects of LY30 on receptors and cFLIP.

**LY30-induced downregulation of cFLIP.** The phenomenon of cFLIP down-regulation upon LY30 treatment was modeled initially (prior to Fig.7) using LY30-induced FLIP degradation as follows: $FLIP + LY30 \xrightarrow{k37} LY30$, where the parameter *k_37* was

estimated so that the simulated level of FLIP declined at 3 hrs to match the fold-change observed at 3hrs in our previously published experiments (Poh et al, 2007).

**Caspase Rates.** Non-specific reactions for synthesis and degradation were added manually with the rates specified. Then automated parameter optimization was performed with the KroneckerBio Toolbox, varying the reaction rates for the caspase enzymes, so that simulations of LY30 alone and simulations of TRAIL alone would match previously published time kinetics of Caspase-3 and Caspase-8 activity after treatment with LY30 alone and TRAIL alone (Poh et al, 2007).

**LY30-induced ROS production.** As we did not know the exact mechanism behind the ROS production upon LY30 treatment, so we simulated a simplistic pseudo-reaction in which LY30 induces ROS production, $LY30 \xrightarrow{k35} LY30 + ROS$. To implement a delay of ROS effect in the pathway, we added another reaction $ROS \underset{k\_36}{\overset{k36}{\rightleftarrows}} ROS^*$ where ROS is an ineffective intermediate state and ROS* represents effective ROS that can contribute to apoptosis. The reaction rates for ROS to cause apoptosis or mitochondrial permeabilization were adjusted manually, to maximize the agreement between the Monte Carlo population simulation of viability, and the observed viability, as a fold-change from untreated to LY30-treated conditions.

*Supplementary Figure 1.5: Single Cells versus Cell Populations (in silico)*

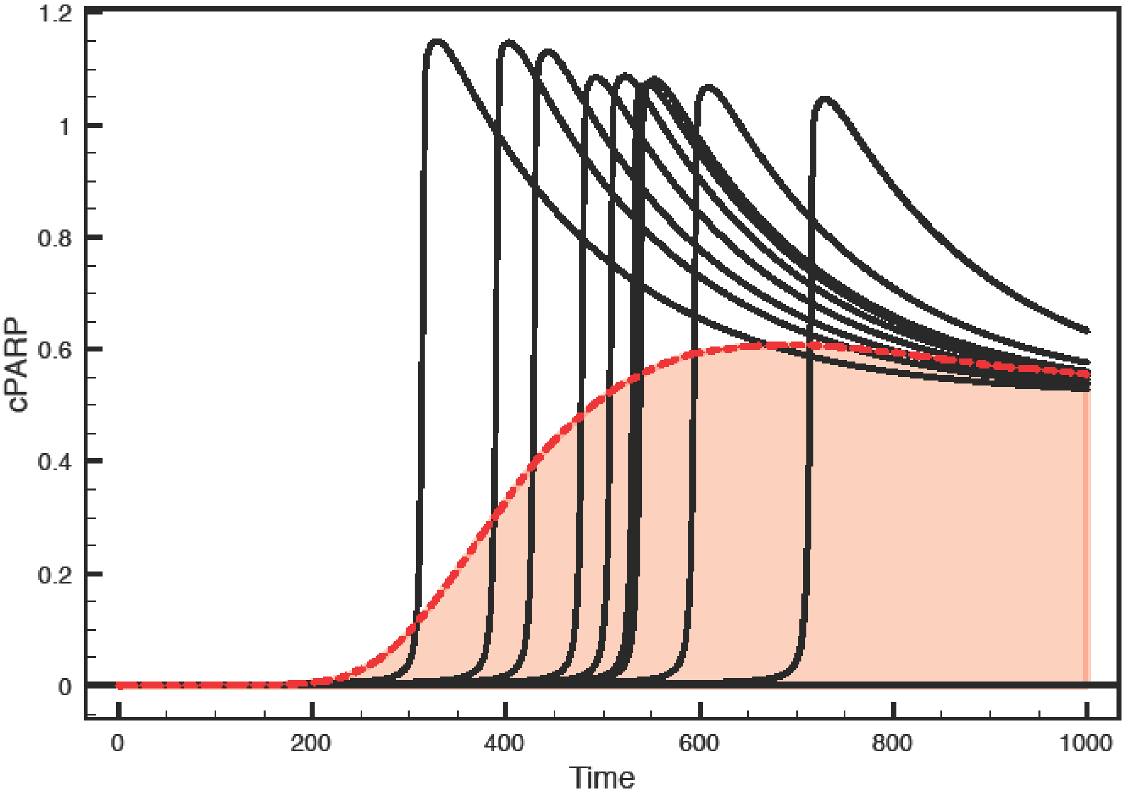


Supplementary Figure 1.5: Comparison of simulation profiles between single cells and a cell population. The x-axis is time after adding drug, and the y-axis is the relative concentration of cPARP. In this figure, each solid line represents one single cell and the dotted line with orange filing is the averaged result of 10,000 single cells.

### *Supplementary Text 1.6: Conversion of absolute activity into relative fold-change*

The model is simulated using absolute protein levels, but Figure 4 shows simulations plotted as predictions of experimental observations in terms of relative enzyme activity. The caspase family of cysteine proteases can target different substrates according to structure. Caspase-8 activity is typically measured as the cleavage of the peptide IETD (Ile-Glu-Thr-Asp), using the fluorescence of Ac-IETD-AFC. Caspase-3 activity is typically measured as the cleavage of DEVD, using the fluorescence of Ac-Asp-Glu-Val-Asp-pNA. Fluorescent substrates were purchased from (BioMol, Plymouth Meeting, PA, USA) and used according to manufacturer's instructions. McStay et al. found that commercial caspase substrates, such as the ones we used, are not entirely specific for their respective cysteine proteases (McStay, et al., 2008), and there is overlapping cleavage motif selectivity. We converted our simulations of absolute protein levels into hypothetical IETD and DEVD fluorescence using a weighted combination of individual caspase levels, according to the quantification of overlap in (McStay, et al., 2008). *IETD_abs* (*DEVD_abs*) refers to absolute IETD (DEVD) fluorescence and *IETD_rel* (*DEVD_rel*) refers to relative IETD (DEVD) fluorescence in treated cells versus untreated cells.

$$IETD\_abs = 0.026 * C8 + 0.8 * C8^{*} + 0.015 * C9^{*}$$

$$IETD\_rel = {IETD\_abs}\Big/{IETD\_abs_{untreated}}$$

$$DEVD\_abs = 30.04 * C3^{*} + 0.068 * C3 + 0.07 * C9^{*}$$

$$DEVD\_rel = {DEVD\_abs}\Big/{DEVD\_abs_{untreated}}$$

***Supplementary Figure 1.7: Caspase-3 activity in experiments and simulations.***

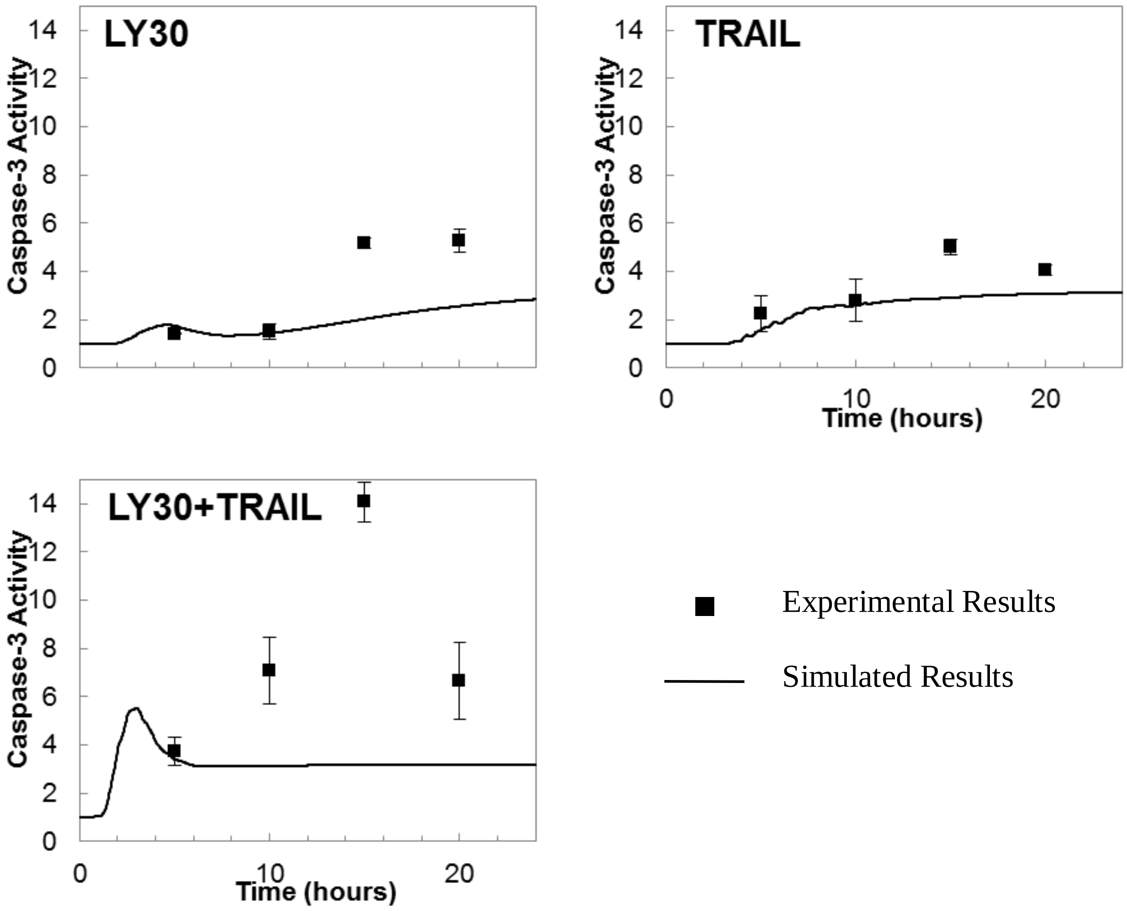

## *Supplementary Figure 1.8: Simulated Trajectories of Species*

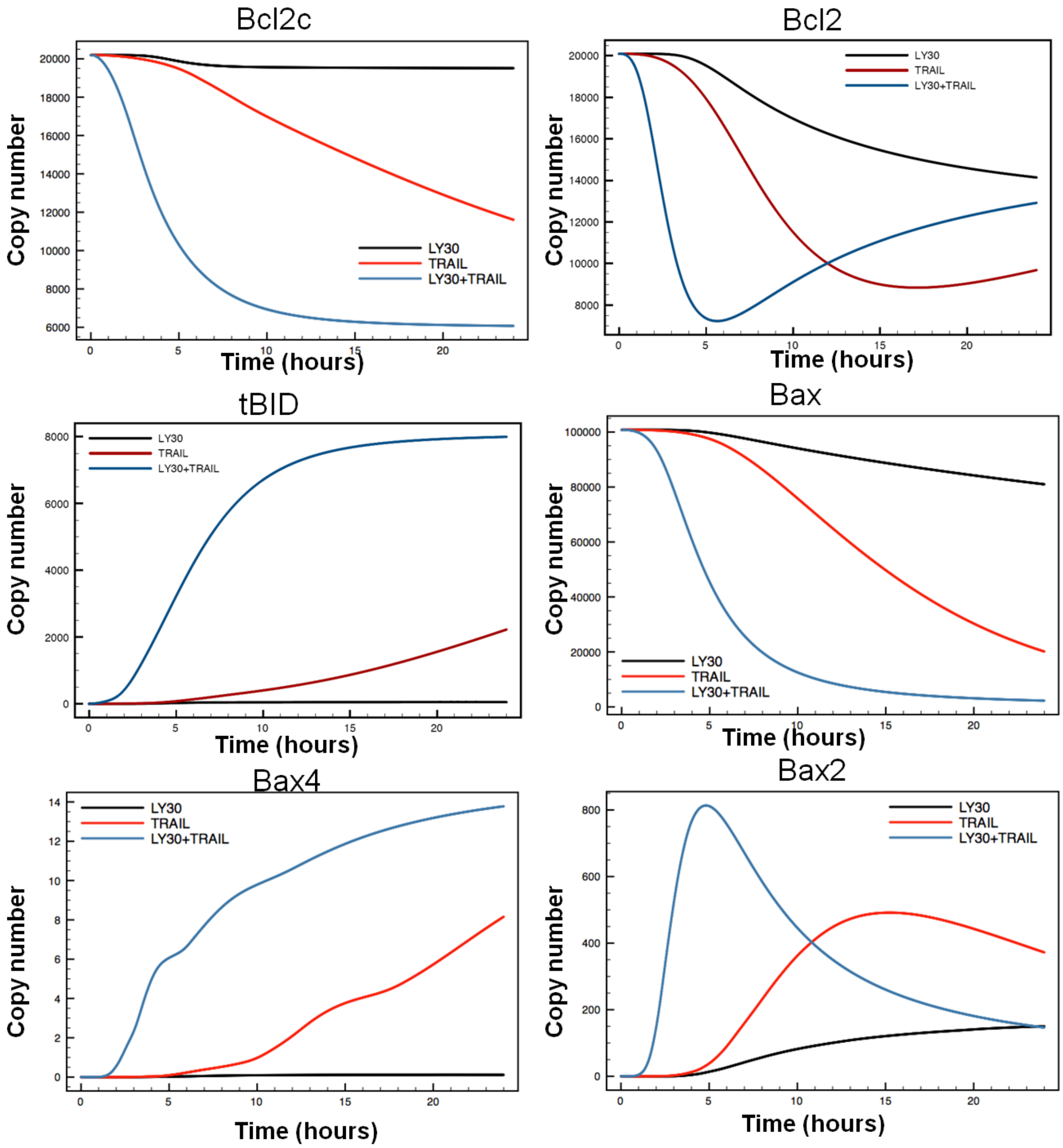

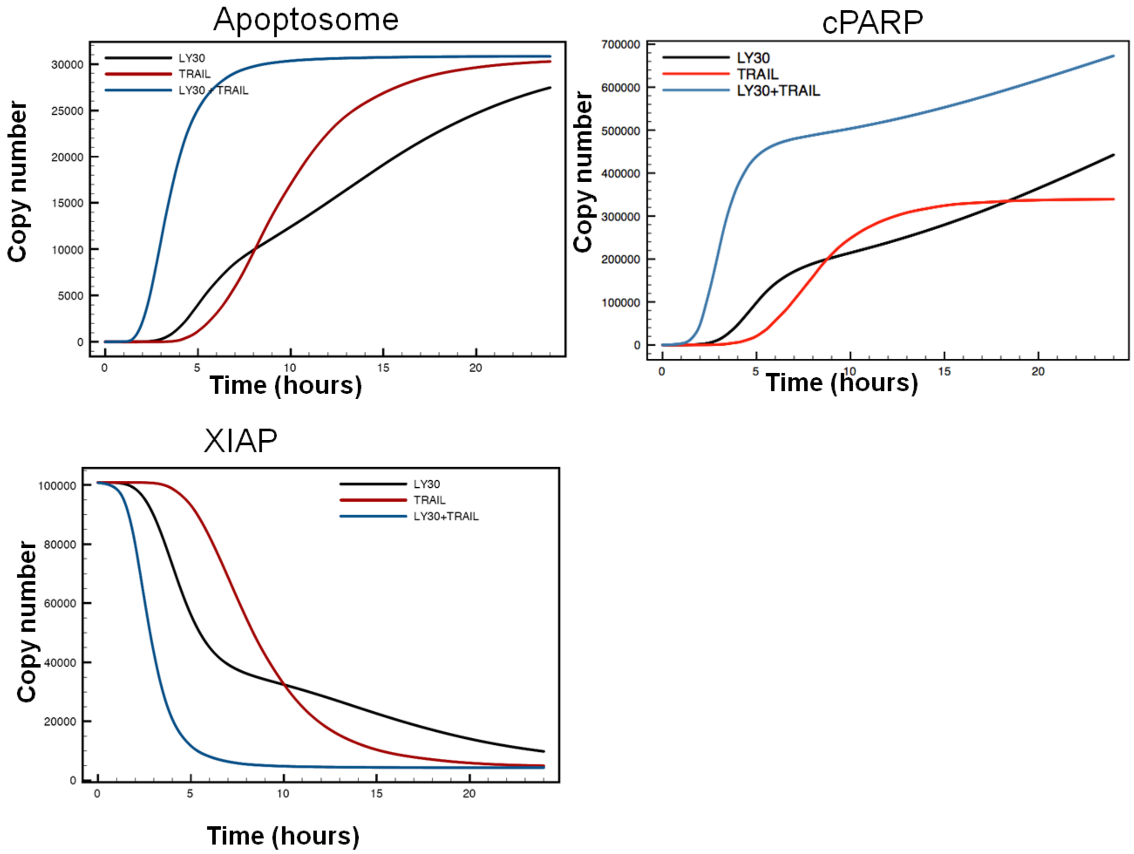

Apoptosome
LY30
TRAIL
LY30 + TRAIL
Copy number
Time (hours)
cPARP
LY30
TRAIL
LY30+TRAIL
Copy number
Time (hours)
XIAP
LY30
TRAIL
LY30+TRAIL
Copy number
Time (hours)

# 2. The ROS-cFLIP Model

## *Supplementary Table 2.1: Reactions of the ROS-cFLIP model*

$$CoQ^- + O_2 \underset{k\_1}{\overset{k1}{\rightleftarrows}} COQ + O_2^-$$

$$Mn_3SOD + O_2^- \underset{k\_2}{\overset{k2}{\rightleftarrows}} Mn_3SOD : O_2^-$$

$$Mn_3SOD : O_2^- \underset{k\_3}{\overset{k3}{\rightleftarrows}} Mn_2SOD + O_2$$

$$Mn_2SOD + O_2^- \underset{k\_4}{\overset{k4}{\rightleftarrows}} Mn_2SOD : O_2^-$$

$$Mn_2SOD : O_2^- + 2H^+ \underset{k\_5}{\overset{k5}{\rightleftarrows}} Mn_3SOD + H_2O_2$$

$$Mn_2SOD : O_2^- \underset{k\_6}{\overset{k6}{\rightleftarrows}} DEP$$

$$2H^+ + 2O_2^- \underset{k\_7}{\overset{k7}{\rightleftarrows}} O_2 + H_2O_2$$

$$GPX_r + H_2O_2 + H^+ \underset{k\_8}{\overset{k8}{\rightleftarrows}} GPX_0 + H_2O$$

$$GPX_0 + GSH \underset{k\_9}{\overset{k9}{\rightleftarrows}} GSGP_x + GSSG + H_2O$$

$$GSGP_x + GSH \underset{k\_10}{\overset{k10}{\rightleftarrows}} GPX_r + GSSG + H^+$$

$$2GSH \underset{k\_11}{\overset{k11}{\rightleftarrows}} GSSG$$

$$CoQ^- + O_2^- + 2H^+ \xrightarrow{k12} CoQ + H_2O_2$$

$$CoQ \underset{k\_13}{\overset{k13}{\rightleftarrows}} CoQ^-$$

$$O_2 \underset{k\_14}{\overset{k14}{\rightleftarrows}} \phi$$

$$LY30 + CoQ \underset{k\_15}{\overset{k15}{\rightleftarrows}} LY30 + CoQ^-$$

$$cFLIP + DEG\_catalyst \xrightarrow{k16} cFLIP\_Deg + DEG\_catalyst$$

$$Deg\_catalyst + O_2^- \underset{k\_17}{\overset{k17}{\rightleftarrows}} Deg\_catalyst : O_2^-$$

$$H_2O_2 + cFLIP_{promoter} \underset{k\_18}{\overset{k18}{\rightleftarrows}} H_2O_2 : cFLIP_{promoter}$$

$$cFLIP_{mRNA} \xrightarrow{k19} cFLIP + cFLIP_{mRNA}$$

$$cFLIP_{promoter} \xrightarrow{k20} cFLIP_{mRNA} + cFLIP_{promoter}$$

$$cFLIP_{promoter} \xrightarrow{k21} \phi$$

$$cFLIP_{mRNA} \xrightarrow{k22} \phi$$

This ROS-cFLIP model is intended to represent multiple possible sources of ROS including NADPH oxidase. However, for specificity of kinetics, the reactions describe ROS production by mitochondrial respiratory chain complexes (Buettner, et al., 2006).

ROS production from other sources might have qualitatively similar dynamics with different kinetic details.

### *Supplementary Table 2.2: Initial Concentrations of the ROS-cFLIP Model*

| Species | Concentration(mol/$m^3$) |
|---|---|
| $O_2$ | 0.000025 |
| $O_2^-$ | 1.23817E-11 |
| $Mn_3SOD$ | 0.000001 |
| $Mn_2SOD$ | 0.000001 |
| $Mn_3SOD{:}O_2^-$ | 3.09539E-13 |
| $Mn_2SOD{:}O_2^-$ | 3.09539E-13 |
| DEP | 2.012E-11 |
| $GPX_0$ | 1.97351E-10 |
| $GPX_r$ | 0.000001 |
| $H^+$ | 1 |
| $H_2O$ | 55 |
| $GSGP_x$ | 7.89405E-13 |
| GSH | 0.001 |
| GSSG | 9.61895E-06 |
| CoQ | 0.00045 |
| $CoQ^-$ | 0.0000001 |
| $H_2O_2$ | 3.68748E-10 |

| | |
|---|---|
| cFLIP | 3.92335E-06 |
| cFLIP_deg | 1 |
| DEG_catalyst | 6.17633E-16 |
| DEG_catalyst:O2- | 3.82367E-16 |
| $cFLIP_{mRNA}$ | 7.06225E-14 |
| $cFLIP_{promoter}$ | 3.53096E-16 |
| LY30 | 1 |
| $H_2O_2$:$cFLIP_{promoter}$ | 6.46904E-16 |

***Supplementary Table 2.3: Reaction Rates of the ROS-cFLIP model***

| Parameter | value | Reference |
|---|---|---|
| k1 | 8000 | (Audi, et al., 2003) |
| k_1 | 800000 | (Audi, et al., 2003) |
| k2 | 1500000000 | (Abreu, et al., 2008; Flohe, et al., 1972) |
| k_2 | 35000 | (Abreu, et al., 2008) |
| k3 | 25000 | (Abreu, et al., 2008) |
| k_3 | 0 | (Abreu, et al., 2008) |
| k4 | 1500000000 | (Abreu, et al., 2008; Privalle and Fridovich, 1990) |
| k_4 | 35000 | (Abreu, et al., 2008) |
| k5 | 25000 | (Abreu, et al., 2008) |
| k_5 | 300 | (Abreu, et al., 2008) |
| k6 | 650 | (Abreu, et al., 2008) |
| k_6 | 10 | (Abreu, et al., 2008) |
| k7 | 240000 | (Lee, 2006) |
| k_7 | 0 | (Lee, 2006) |
| k8 | 21000000 | (Flohe, et al., 1972) |
| k_8 | 0 | (Flohe, 1978; Flohe, et al., 1972) |
| k9 | 40000 | (Flohe, 1978; Flohe, et al., 1972) |
| k_9 | 0 | (Flohe, 1978; Flohe, et al., 1972) |
| k10 | 10000000 | (Flohe, 1978; Flohe, et al., 1972) |
| k_10 | 0 | (Flohe, et al., 1972) |
| k11 | 10000 | (Buettner, et al., 2006) |
| k_11 | 1000 | (Buettner, et al., 2006) |

| | | |
|---|---|---|
| k12 | 3000000 | (Buettner, et al., 2006) |
| k13 | 100 | Estimated from (Buettner, et al., 2006) |
| k_13 | 450000 | Estimated from (Buettner, et al., 2006) |
| k14 | 0.0000025 | Estimated |
| k_14 | 0.1 | Estimated |
| K15 | 1000 | Estimated |
| K_15 | 10 | Estimated |
| k16 | 2.92E+12 | Estimated |
| k17 | 50000000000 | Estimated |
| k_17 | 1 | Estimated |
| k18 | 1000000 | Estimated |
| k_18 | 0.0002 | Estimated |
| k19 | 10 | Estimated |
| k20 | 100000 | Estimated |
| k21 | 100 | Estimated |
| k22 | 0.05 | Estimated |

# 3. The TRAIL+ LY30 Model Combined with ROS-cFLIP

## *Supplementary Figure 3.1: the TRAIL+ LY30 model combined with ROS-cFLIP*

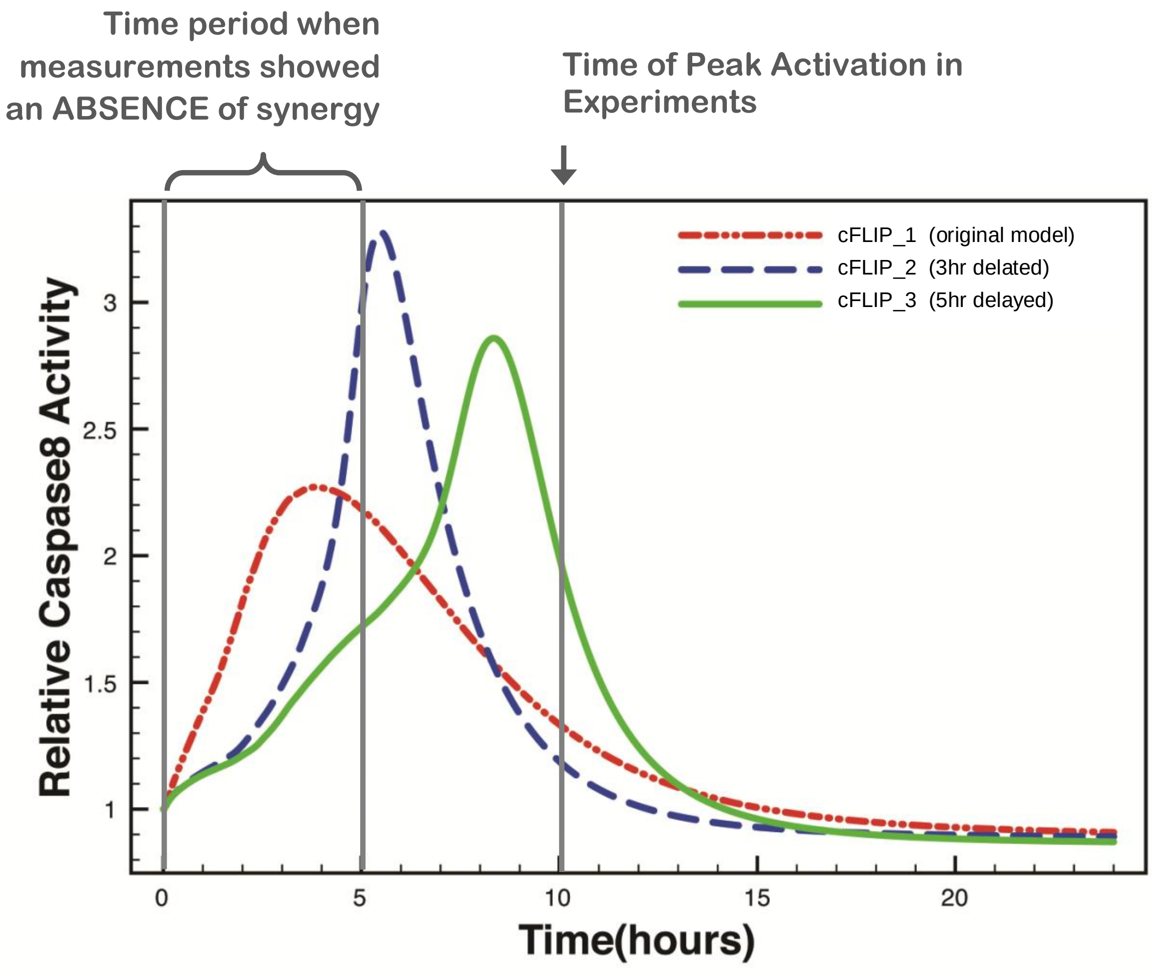


Supplementary Figure 3.1a: Simulations the TRAIL+LY30 model showing caspase-8 after simulation with LY+T, using different cFLIP trajectories as input. cFLIP trajectories appear in Supplementary Fig 3.1b. The red dashed curve shows caspase-8 levels after treatment with LY+T, when cFLIP decays immediately after LY30 treatment. Delayed cFLIP can decrease the appearance of synergy in caspase-8 activation at 0-5 hrs. A thick grey line at 10 hrs marks when experiments observed peak activation of caspase-8 (Figure 4 and Supplementary Figures 4.2-4.3). The cFLIP trajectory would have to be unreasonably delayed (green curve, “Simulated cFLIP_3 delayed 5hr”) to cause the caspase-8 peak to occur at 8.5 hrs (“Simulated cFLIP_2”).

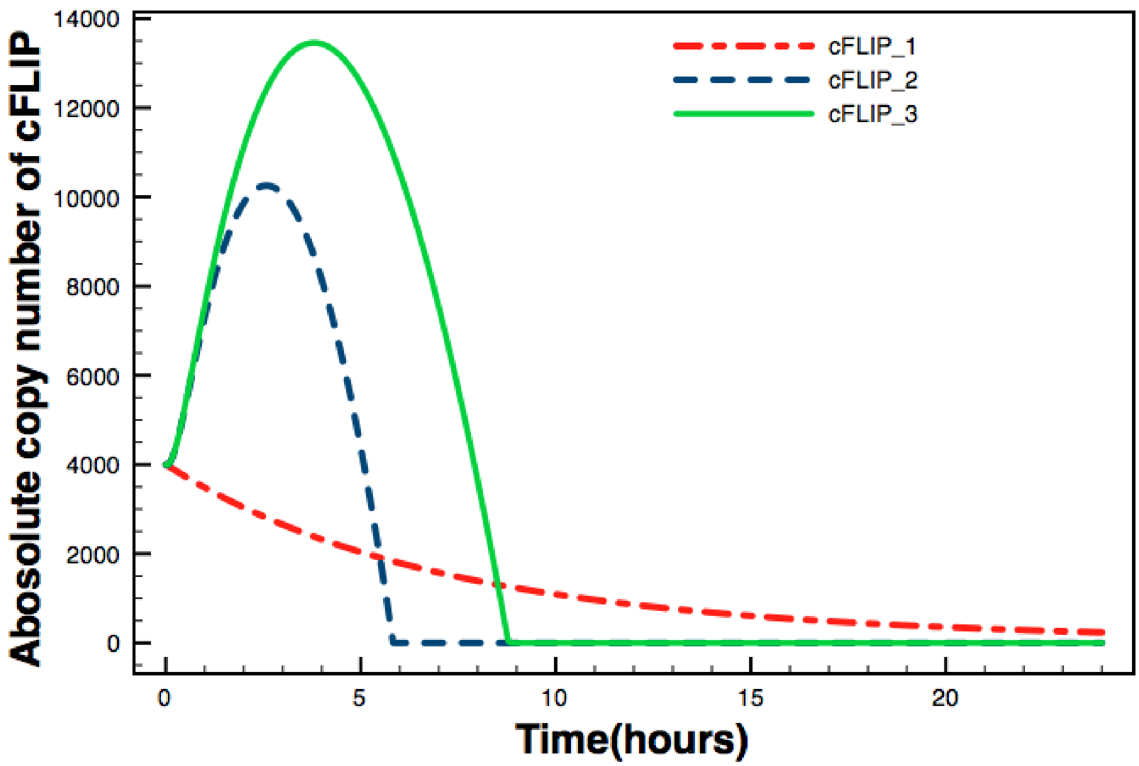


Supplementary Figure 3.1b: cFLIP trajectories used when simulating Supplementary Figure 3.1a. In the red dashed curve, cFLIP decays immediately after LY30 treatment. The cFLIP trajectory would have to be delayed artificially to cause the delay in the caspase-8 peak seen in Figure 3.1a.

# 4. Biological Results

## *Supplementary Text 4.1: Supplementary Experimental Methods*

**Cell transfection.** HeLa cells (seeded at $10^6$ cells per 100mm Petri dishes the day before) were transfected with 5µg of the empty plasmid pcDNA3-neo (Invitrogen, Carlsbad, CA) or a plasmid containing the full-length human bcl-2 gene pcDNA3-Bcl-2 using the calcium phosphate method. Briefly, 5µg of DNA were diluted in 500µL of a 125mM $CaCl_2$ solution. Then, 500µL of 2xHBS solution (50mM HEPES ph 7.05, 10mM KCl, 280mM NaCl, 1.5mM $Na_2PO_4$) was added drop-wise to the DNA solution while vortexing at low speed. The transfection mix was then incubated at room temperature for 30 minutes before being added drop-wise onto the cells in antibiotic-free medium. Cells were cultured for 24 hours before trypsinization and seeding for subsequent experiments.

**ROS measurement.** HeLa cells were pre-incubated with 10mM Tiron for 2 hours or with 2000U/mL catalase overnight. After being pre-loaded with 5µM dihydroethidium (DHE) (Molecular Probes, Invitrogen, Carlsbad, CA) and 5µM 5-(and-6)-chloromethyl-2',7'-dichlorodihydrofluorescein diacetate, acetyl ester (CM-DCFDA) (Molecular Probes, Invitrogen, Carlsbad, CA) for 30 minutes in plain DMEM, cells were trypsinized and resuspended in plain DMEM in the presence or absence of 25µM LY30 for 5 or 15 minutes. Fluorescence was measured using a TECAN Infinite M200 microplate reader (TECAN trading AG, Switzerland) (DHE: 488nm excitation / 585 nm emission, DCFDA: 492nm excitation / 520 emission).

**Antibodies.** Mouse monoclonal cFLIP antibody (Alexis Biochemicals, Enzo Life Science, Exeter, UK). Mouse monoclonal FADD antibody (BD Pharmingen, San Diego, CA, USA). Mouse monoclonal Bcl-2 antibody (Santa Cruz Biotechnology Inc., Santa Cruz, CA)

*Supplementary Figure 4.2: Caspase-8 activity in HeLa with empty vector control*

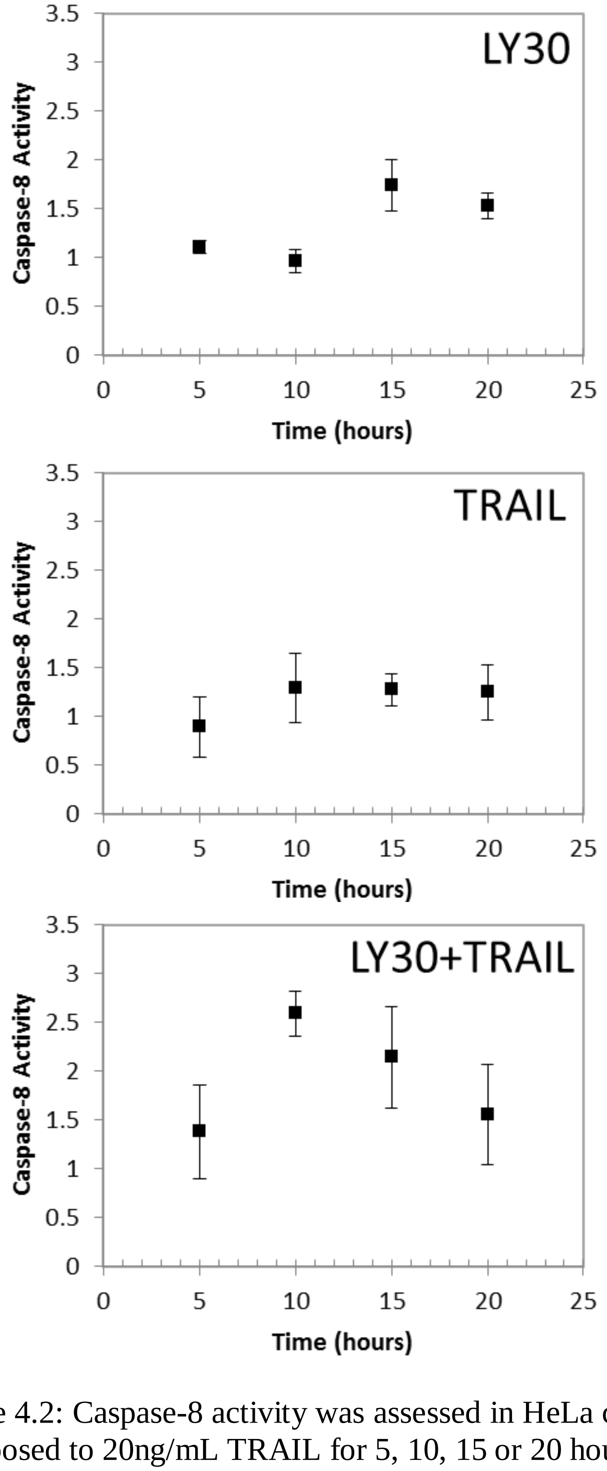


Supplementary Figure 4.2: Caspase-8 activity was assessed in HeLa cells transfected with pcDNA3-neo and exposed to 20ng/mL TRAIL for 5, 10, 15 or 20 hours with or without pre-incubation with 25uM LY30 for 1 hour.

***Supplementary Figure 4.3: Caspase-8 activity in HeLa with Bcl2 overexpression***

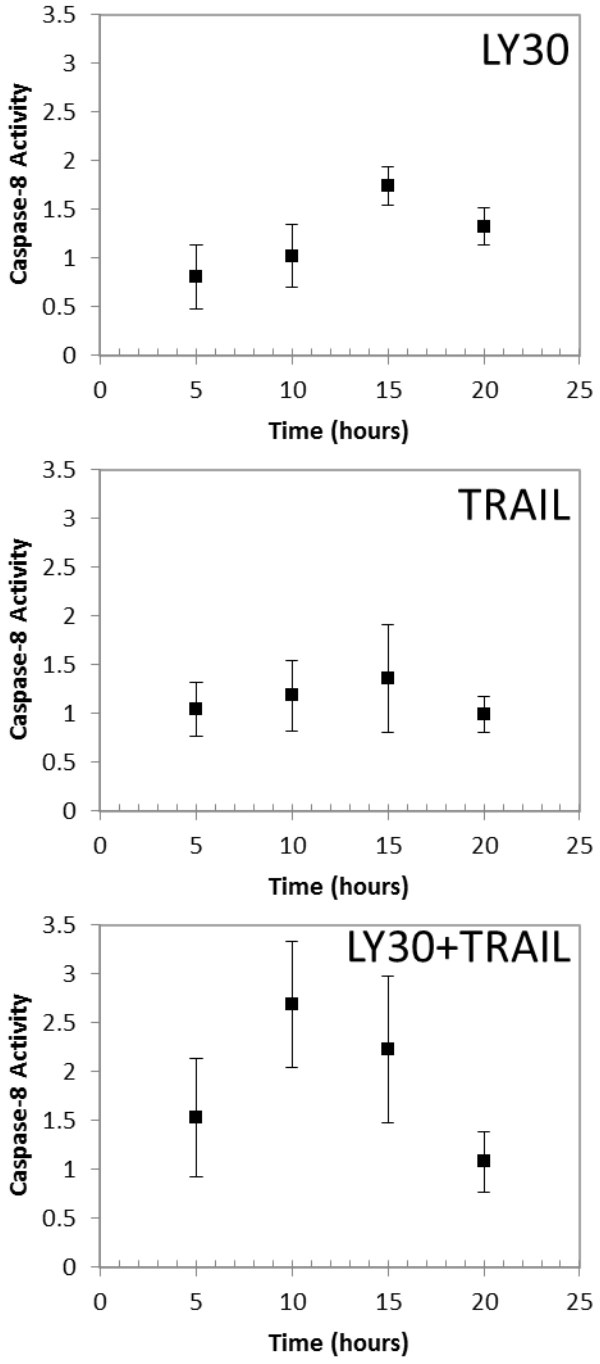


Supplementary Figure 4.3: Caspase-8 activity was assessed in HeLa cells transfected with pcDNA3-Bcl-2 and exposed to 20ng/mL TRAIL for 5, 10, 15 or 20 hours with or without pre-incubation with 25uM LY30 for 1 hour.

***Supplementary Figure 4.4: Caspase-3 activity in HeLa with empty vector control***

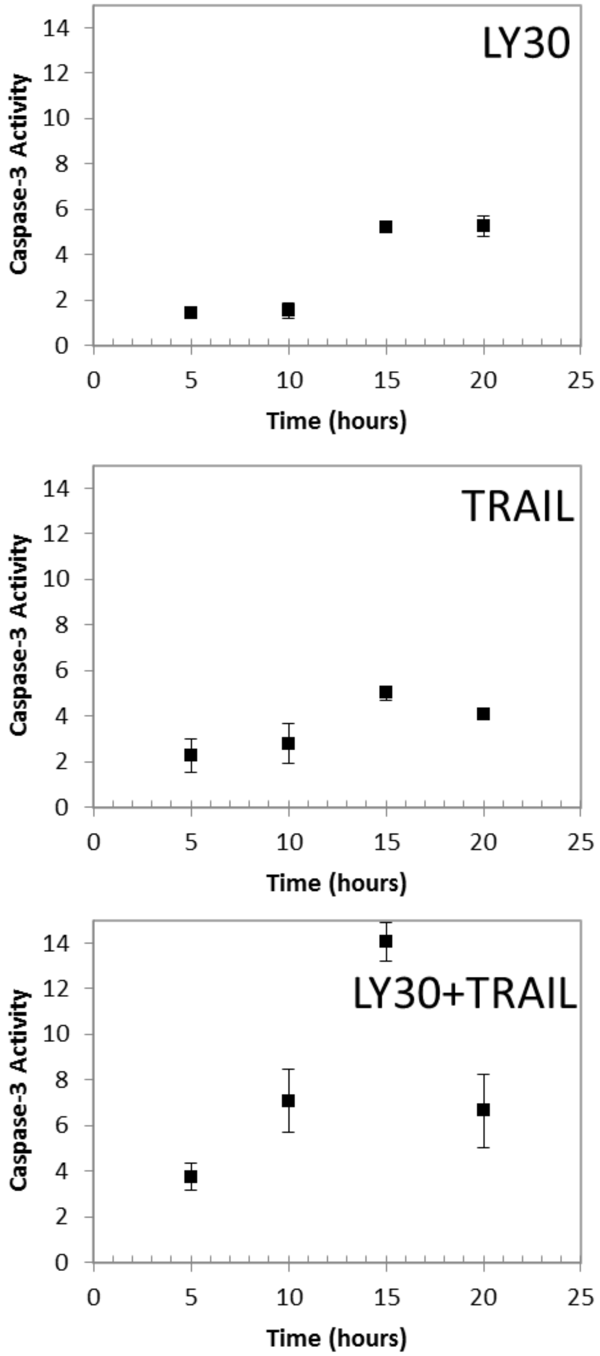


Supplementary Figure 4.4: Caspase-3 activity was assessed in HeLa cells transfected with pcDNA3-neo and exposed to 20ng/mL TRAIL for 5, 10, 15 or 20 hours with or without pre-incubation with 25μM LY30 for 1 hour.

***Supplementary Figure 4.5: Caspase-3 activity in HeLa with Bcl2 overexpression***

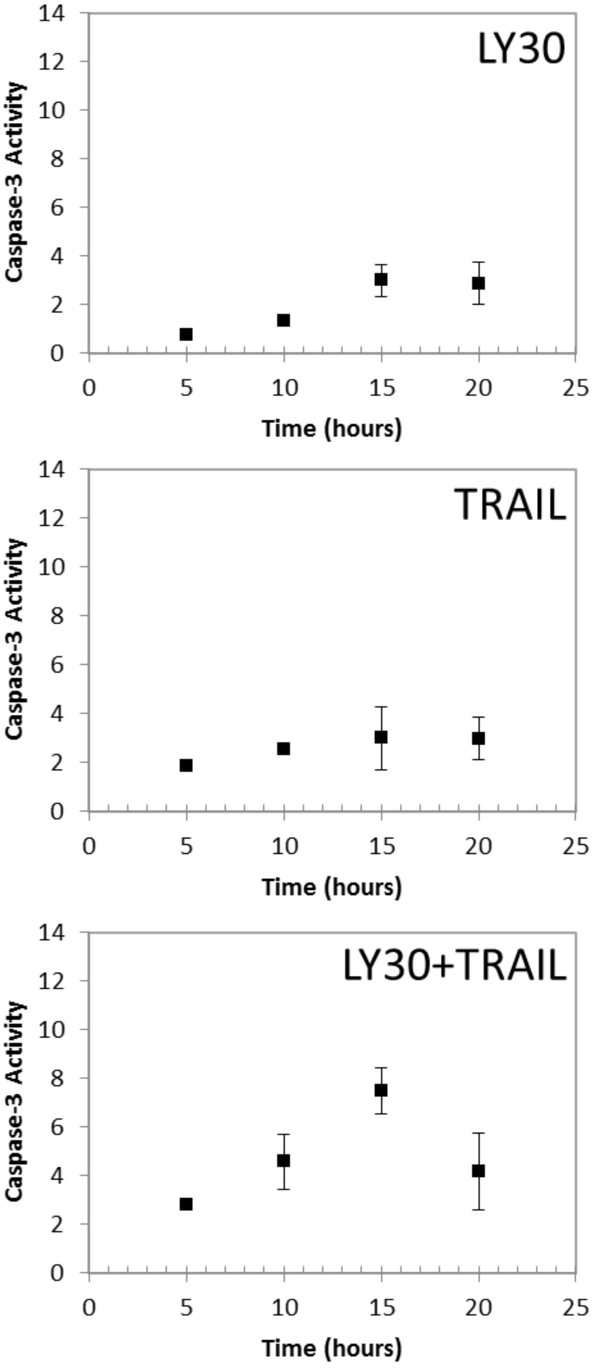


Supplementary Figure 4.5: Caspase-3 activity was assessed in HeLa cells transfected with pcDNA3-Bcl-2 and exposed to 20ng/mL TRAIL for 5, 10, 15 or 20 hours with or without pre-incubation with 25uM LY30 for 1 hour.

*Supplementary Figure 4.6: LY30-induced changes in cFLIP levels*

(a)

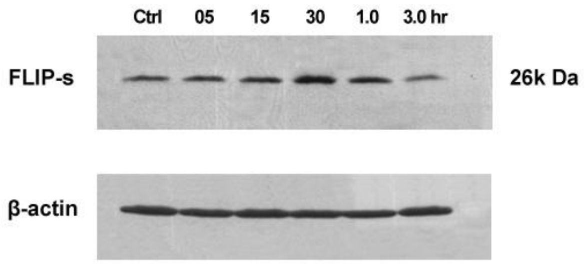


(b)

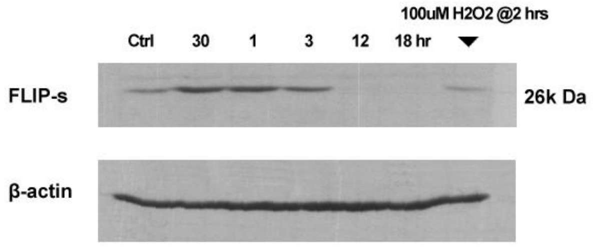


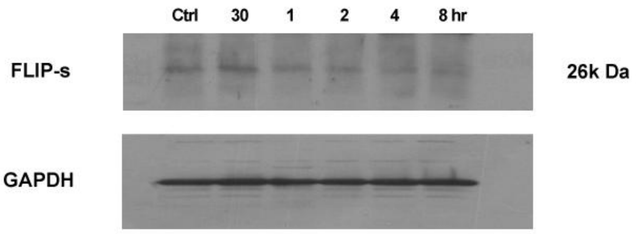


(c)

Supplementary Figure 4.6. Hela cells ($1X10^5$) were treated with 25μM of LY30 for the times indicated (5min-18hr) and whole cell lysates were then used in Western blots for cFLIP. β-actin or GAPDH were used as a loading controls.

***Supplementary Figure 4.7: FADD expression.***

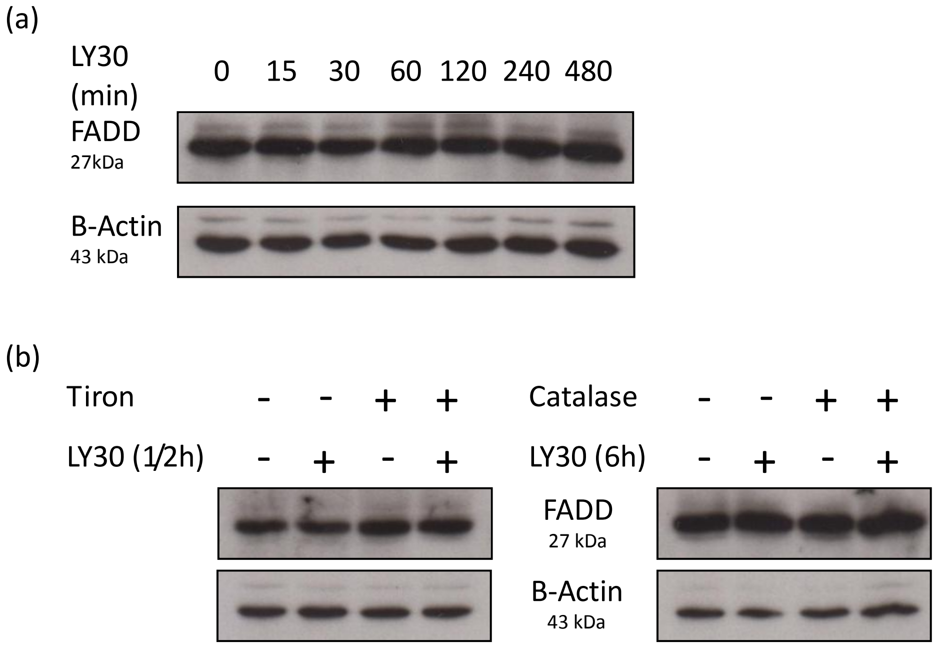


Supplementary Figure 4.7: FADD expression. (a) Western blot analysis of FADD in HeLa after different durations of LY30 treatment; (b) Western blot analysis of FADD in HeLa in the absence or presence of ROS scavengers Tiron or catalase. HeLa cells are pre-incubated with 10mM Tiron for 2 hour before addition of 25μM LY30 for 30 minutes or pre-incubated with 2000U/mL catalase overnight and co-incubated with 25μM LY30 for 6 hours.

## *Supplementary Figure 4.8: Activity of ROS Scavengers*

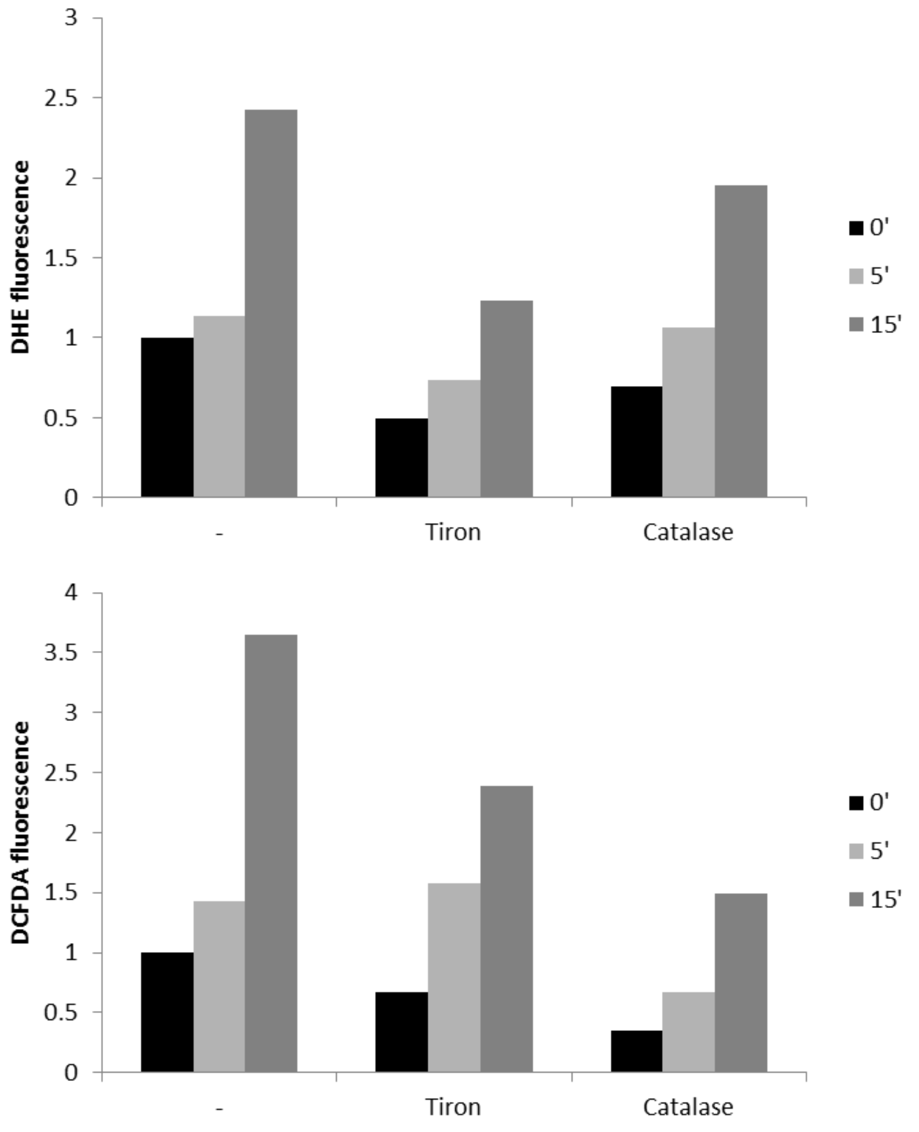


Supplementary Figure 4.8. ROS measurement. (a) Dihydroethidine (DHE) is used as an indicator for superoxide anion. (b) Staining with DCFDA to indicate ROS in HeLa cells treated with 25uM LY30 for 5 or 10 minutes in the presence or absence of ROS scavengers. 10mM Tiron was pre-incubated for 2 hours before the addition of LY30 while 2000U/mL catalase was pre-incubated overnight and then co-incubated with LY30. The indicator DCFDA is strongly activated by hydrogen peroxide and not by superoxide, but DCFDA can also be activated by highly reactive species such as hydroxyl radical. Quantification of the specificity has been published (Molecular Probes, The Handbook, Invitrogen).

# 5. REFERENCES


Abreu, I.A., Hearn, A., An, H., Nick, H.S., Silverman, D.N. and Cabelli, D.E. (2008) The kinetic mechanism of manganese-containing superoxide dismutase from Deinococcus radiodurans: a specialized enzyme for the elimination of high superoxide concentrations, *Biochemistry*, **47**, 2350-2356.

Albeck, J.G., Burke, J.M., Aldridge, B.B., Zhang, M., Lauffenburger, D.A. and Sorger, P.K. (2008) Quantitative analysis of pathways controlling extrinsic apoptosis in single cells, *Molecular cell*, **30**, 11-25.

Audi, S.H., Zhao, H., Bongard, R.D., Hogg, N., Kettenhofen, N.J., Kalyanaraman, B., Dawson, C.A. and Merker, M.P. (2003) Pulmonary arterial endothelial cells affect the redox status of coenzyme Q0, *Free Radic Biol Med*, **34**, 892-907.

Buettner, G.R., Ng, C.F., Wang, M., Rodgers, V.G. and Schafer, F.Q. (2006) A new paradigm: manganese superoxide dismutase influences the production of H2O2 in cells and thereby their biological state, *Free Radic Biol Med*, **41**, 1338-1350.

Delmas, D., Rebe, C., Micheau, O., Athias, A., Gambert, P., Grazide, S., Laurent, G., Latruffe, N. and Solary, E. (2004) Redistribution of CD95, DR4 and DR5 in rafts accounts for the synergistic toxicity of resveratrol and death receptor ligands in colon carcinoma cells, *Oncogene*, **23**, 8979-8986.

Flohe, L. (1978) Glutathione peroxidase: fact and fiction, *Ciba Found Symp*, 95-122.

Flohe, L., Loschen, G., Gunzler, W.A. and Eichele, E. (1972) Glutathione peroxidase, V. The kinetic mechanism, *Hoppe Seylers Z Physiol Chem*, **353**, 987-999.

Lee, B.G.K.J.H. (2006) Determination of Hydroperoxyl/superoxide Anion Radical (HO2·/O2·−) Concentration in the Decomposition of Ozone Using a Kinetic Method, *Bull. Korean Chem. Soc.*, **27**.

McStay, G.P., Salvesen, G.S. and Green, D.R. (2008) Overlapping cleavage motif selectivity of caspases: implications for analysis of apoptotic pathways, *Cell Death Differ*, **15**, 322-331.

Mellier, G. and Pervaiz, S. (2012) The three Rs along the TRAIL: resistance, re-sensitization and reactive oxygen species (ROS), *Free Radic Res*, **46**, 996-1003.

Poh, T.W., Huang, S., Hirpara, J.L. and Pervaiz, S. (2007) LY303511 amplifies TRAIL-induced apoptosis in tumor cells by enhancing DR5 oligomerization, DISC assembly, and mitochondrial permeabilization, *Cell death and differentiation*, **14**, 1813-1825.

Poh, T.W., Huang, S., Hirpara, J.L. and Pervaiz, S. (2007) LY303511 amplifies TRAIL-induced apoptosis in tumor cells by enhancing DR5 oligomerization, DISC assembly, and mitochondrial permeabilization, *Cell Death Differ*, **14**, 1813-1825.

Privalle, C.T. and Fridovich, I. (1990) Anaerobic biosynthesis of the manganese-containing superoxide dismutase in Escherichia coli. Effects of diazenedicarboxylic acid bis(N,N'-dimethylamide) (diamide), *J Biol Chem*, **265**, 21966-21970.
